# Transformer-Based Multiomics Study Identifies Important Role of Glycine, Serine, and Threonine Metabolism Pathway in Rheumatoid Arthritis Complicated by Anemia

**DOI:** 10.34133/csbj.0075

**Published:** 2026-05-07

**Authors:** Jiaxin Huang, Yuanli Wei, Dongmei Wang, Zhuming Yin, Jianghua Chen, Congcong Jian, Xiaoting Zhu, Shilin Li, Jie Zhang, Tingting Wang, Caizhen Liu, Lingli Wei, Jing Gao, Jing Zhu, Qinghua Zou, Jianhong Wu, Fanxin Zeng

**Affiliations:** ^1^ Institute of Basic Medicine and Forensic Medicine, North Sichuan Medical College, Nanchong, Sichuan 637000, China.; ^2^ Department of Rheumatology and Immunology, Dazhou Central Hospital, Dazhou, Sichuan 635000, China.; ^3^ Tianjin Medical University Cancer Institute and Hospital, Tianjin 300000, China.; ^4^ Dazhou Vocational College of Chinese Medicine, Dazhou, Sichuan 635000, China.; ^5^ Department of Clinical Research Center, Dazhou Central Hospital, Dazhou, Sichuan 635000, China.; ^6^ Department of Rheumatology and Immunology, Sichuan Provincial People’s Hospital, Chengdu, Sichuan 610072, China.; ^7^ Department of Rheumatology and Immunology, First Affiliated Hospital of Army Medical University, Chongqing 400038, China.; ^8^ Sichuan Provincial Clinical Research Center of Radiology, Dazhou, Sichuan 635000, China.

## Abstract

Rheumatoid arthritis (RA) is frequently complicated by anemia, which exacerbates disease progression and worsens clinical outcomes. This study aimed to characterize the molecular alterations associated with RA complicated by anemia (RA_ane) using a transformer-based machine learning architecture combined with multiomics aggregation. Retrospective clinical analysis demonstrated a high incidence of anemia in patients with RA, which strongly correlated with systemic inflammation (*P* < 0.001). Specifically, hemoglobin levels showed significant negative correlations with C-reactive protein and erythrocyte sedimentation rate. To investigate the underlying molecular landscape, we established a multiomics cohort comprising 113 healthy controls, 257 patients with RA, 88 patients with systemic lupus erythematosus, and 37 patients with gout. By aggregating untargeted metabolomic, microbiomic, and transcriptomic profiles and applying an algorithmic consensus across multiple machine learning models, we identified a reliable panel of RA_ane biomarkers. These included depleted metabolites (e.g., l-tryptophan and glyceric acid) and up-regulated genes (e.g., *ALAS2*, *CA1*, and *HBB*). Functional enrichment analysis identified the dysregulation of the glycine, serine, and threonine metabolism (GSTM) pathway as a central molecular signature of RA_ane. Furthermore, comparative analyses indicated that these GSTM alterations were specific to RA_ane, distinguishing it from anemia in systemic lupus erythematosus and gout. The core metabolic changes and the up-regulation of *ALAS2* were successfully validated in an independent external cohort and by quantitative polymerase chain reaction. In addition, our diagnostic classifiers demonstrated strong predictive performance (area under the receiver operating characteristic curve up to 0.854). This study maps a multidimensional molecular network centered on the GSTM pathway, providing proof-of-concept insights that may inform future diagnostic approaches and mechanism-driven research for RA-associated anemia.

## Highlights

1. This study used a transformer-based approach to intersecting multiomics signatures, systematically characterizing rheumatoid arthritis (RA) complicated by anemia (RA_ane) and emphasizing the pivotal role of the glycine, serine, and threonine metabolism pathway.
2. Comparative analyses revealed that dysregulation of the glycine, serine, and threonine metabolism pathway is uniquely associated with RA_ane, distinguishing it from anemia in systemic lupus erythematosus or gout.
3. A comprehensive retrospective cohort analysis (4,114 RA versus 100,934 non-RA individuals) demonstrated a significantly elevated incidence of anemia among patients with RA (*P* < 0.001) and a robust inverse correlation between hemoglobin levels and systemic inflammation markers.

## Graphical Abstract

**Figure:**
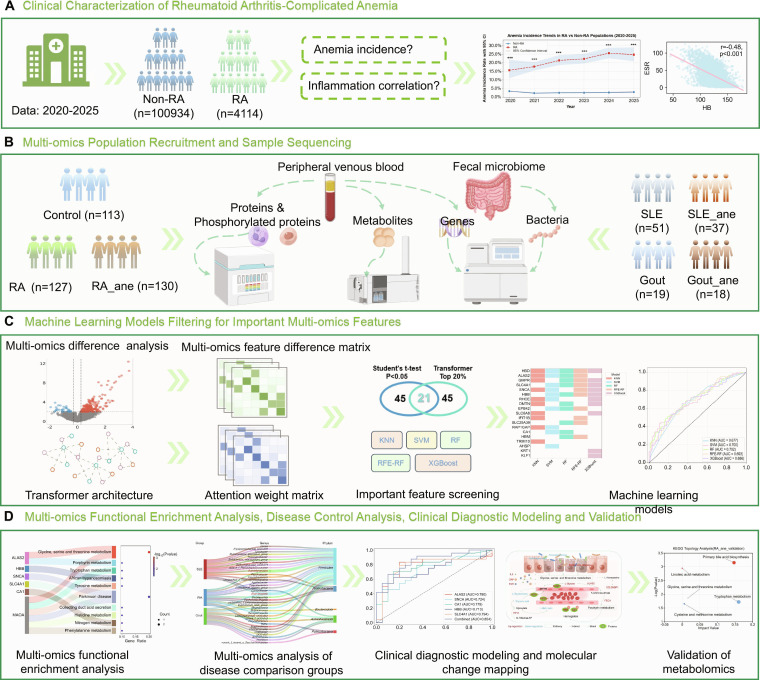

## Introduction

Rheumatoid arthritis (RA) is a common autoimmune inflammatory disease, primarily characterized by chronic synovial inflammation and progressive damage of joints and bones [1,2]. Beyond its effects on the joints, RA markedly impacts the hematological system, with anemia being the most common hematologic manifestation [3]. Approximately 33% to 60% of patients with RA worldwide experience mild anemia, primarily due to chronic disease [4–6]. Numerous studies have demonstrated a significant correlation between the severity of anemia and disease activity in RA, with more severe anemia observed in active disease. Inflammatory mediators such as tumor necrosis factor-α (TNF-α) and interleukin-6 (IL-6) are thought to exacerbate anemia by inhibiting erythropoiesis, increasing red blood cell destruction, and reducing iron availability [7–9]. Furthermore, disease activity and structural joint damage in patients with RA are closely linked to anemia and hemoglobin (HB) levels, with increased disease activity typically correlating with more severe anemia. This positions anemia as a clinically relevant indicator for monitoring RA progression [10,11]. Despite its significance, RA complicated by anemia (RA_ane) is often overlooked in clinical management, and research exploring its targets and mechanisms is limited. Therefore, further investigation into the molecular changes associated with RA_ane is essential for developing diagnostic models and enhancing treatment strategies. In addition, both systemic lupus erythematosus (SLE) and RA share overlapping autoimmune dysfunctions, including the presence of antinuclear antibodies [12], while gout represents a classic crystal-induced autoinflammatory arthritis [13]. Strategically including SLE and gout as disease comparison groups allows us to determine whether the multiomics alterations we observe are specific to RA_ane or merely a generic consequence of pan-inflammatory or pan-autoimmune anemic responses.

Recent advances in comprehensive multiomics approaches have highlighted their potential in the early detection and management of diseases. Artificial intelligence technologies are emerging as revolutionary tools for analyzing the molecular mechanisms of complex diseases by leveraging their high-dimensional data processing and nonlinear pattern recognition capabilities [14,15]. In RA research, multiomics technologies have systematically elucidated the multidimensional features of disease progression. For instance, the synergistic analysis of metabolomics and microbiomics has revealed stage-specific interactions between intestinal dysbiosis and metabolites such as succinic acid and short-chain fatty acids [16]. In addition, the integration of transcriptomics and serum proteomics has yielded quantitative indices for assessing molecular responses of patients with RA to drug therapy [17]. Moreover, diagnostic models based on combined metabolite–microbiome markers have facilitated initial noninvasive assessments of RA activity [18,19]. Although machine learning algorithms, such as extreme gradient boosting (XGBoost) (identifying RA-specific differential metabolites through SHapley Additive exPlanations value interpretations) and recursive feature elimination–random forest (RF) strategy (achieving over 90% prediction accuracy in cross-omics marker screening), have excelled in single-omics biomarker screening [20–23], most existing studies still rely on linear feature extraction approaches. This limits the modeling of nonlinear interaction networks within histological data. In contrast, deep learning architectures, such as transformers, have achieved major breakthroughs in integrating multiomics data for cancer research [24]. The field of RA is yet to establish an intelligent analytical framework that simultaneously captures molecular network dynamics and clinical phenotypic heterogeneity, thereby limiting systematic discovery of precision diagnostic targets.

The transformer architecture substantially enhances multiomics aggregation capabilities by capturing complex nonlinear associations within high-dimensional omics data such as transcriptomics and metabolomics. Unlike traditional deep learning models, transformers leverage multihead self-attention mechanisms to dynamically evaluate the relevance of input features, independent of their spatial positioning. This is crucial for global data, where biological signals may be sparse and exhibit long-range dependencies [25,26]. Attention mechanisms can reveal coordinated transcriptional changes between distant genomic sites affected by gene expression cascades or specific metabolic pathways [27]. This capability facilitates deeper biological insights, such as dissecting subnetworks of specific diseases and identifying collaborative biomarker panels across various omics layers. Recent applications highlight transformers’ effectiveness in tasks such as identifying cancer-driving genes from genomic and proteomic data [28] and enhancing biomarker screening efficiency by modeling long-range dependencies between molecular features [29]. Furthermore, when combined with targeted metabolomics, transformers support discoveries in fields such as pest resistance marker identification by revealing intricate biochemical relationships [30]. In this study, we propose aggregating multiomics analysis with transformer architecture to explore metabolic–transcriptional interactions in RA_ane, aiming to improve diagnostic sensitivity and inform individualized treatment strategies.

## Methods

### Clinical cohorts and multiomics participant recruitment

**Fig. 1.**
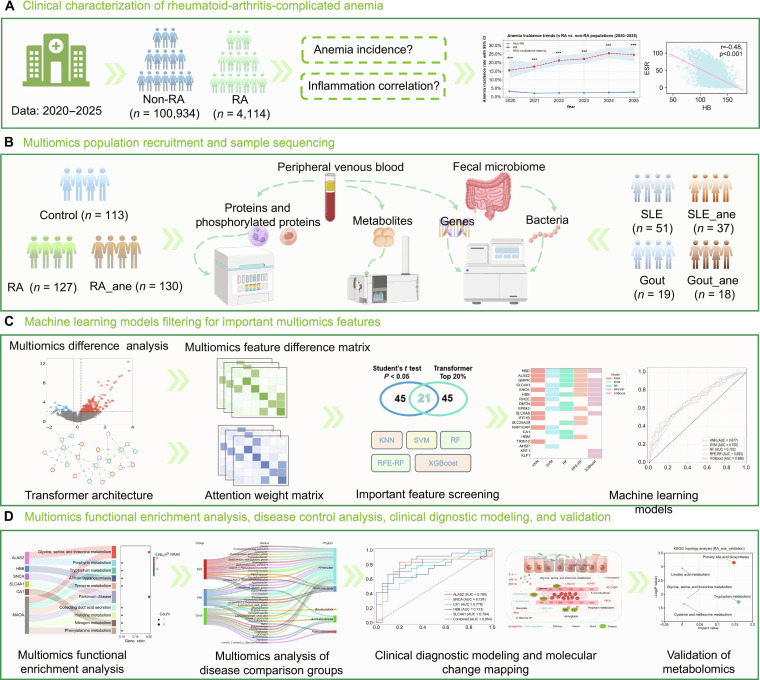
Workflow of analysis in this study. (A) The clinical characteristics of patients with rheumatoid arthritis (RA) complicated by anemia (RA_ane) were analyzed by comparing basic clinical information from 4,114 patients with RA and 100,934 normal individuals (non-RA) at Dazhou Central Hospital between 2020 January and 2025 July. Data were used to assess anemia incidence and its correlation with hemoglobin (HB) and inflammation-related indicators. (B) Population recruitment and sample sequencing: We included 257 patients with RA and 113 controls (medical examiners with nonautoimmune diseases) from Dazhou Central Hospital for multiomics analysis. In addition, 88 patients with systemic lupus erythematosus (SLE) and 37 patients with gout served as disease comparison cohorts. SLE and gout populations were sequenced and analyzed for metabolomics and microbiomics. (C) Machine leaning model-based filtering for key multiomics features: The difference feature matrix was obtained on the basis of multiomics difference analysis, the attention weight matrix of all features was calculated on the basis of the transformer architecture, the intersection of the 2 expression matrices was analyzed to obtain the important difference markers, and 5 machine learning models were used for further feature screening. (D) Multiomics functional enrichment analysis, disease comparisons analysis, clinical diagnostic modeling, and validation: Features identified through the machine learning models were subjected to functional enrichment analysis, followed by validation of the pathways of interest across multiple dimensions, including histological data and comparative disease cohort analysis and an independent external metabolomic cohort. Finally, based on these results, a clinical diagnostic model and multiomics differential map specific to RA_ane were constructed. ***P < 0.001. SLE_ane, SLE complicated by anemia; gout, gouty arthritis; gout_ane, gout complicated by anemia; KNN, *K*-nearest neighbors; SVM, support vector machine; RF, random forest; RFE-RF, recursive feature elimination-RF; XGBoost, extreme gradient boosting; CI, confidence interval.

This study utilized 2 distinct patient populations to independently evaluate the epidemiological incidence and the molecular landscape of RA_ane. First, a large-scale retrospective clinical cohort was established to calculate the incidence of anemia. This epidemiological cohort comprised patients diagnosed with RA at Dazhou Central Hospital between 2020 January 1 and 2025 July 1, alongside a concurrent non-RA control population derived from the Physical Examination Center during the same timeframe. All RA diagnoses fulfilled the 2010 American College of Rheumatology (ACR)/European League Against Rheumatism (EULAR) classification criteria [1]. Second, an independent multiomics cohort was prospectively assembled between 2017 and 2022 to conduct molecular sequencing. Blood and fecal samples were sequentially collected from a total of 495 participants. This molecular analysis cohort comprised 257 patients with RA, 113 healthy controls, and 125 disease controls (88 patients with SLE and 37 patients with gout) (Fig. 1). The patients with SLE and the patients with gout fulfilled the respective 2019 EULAR/ACR and 2015 EULAR/ACR classification criteria [31,32]. Strict and unified inclusion and exclusion criteria were applied across both the epidemiological and multiomics cohorts. Individuals under 18 years of age, as well as those with concurrent malignancies, bleeding disorders, or overlapping systemic autoimmune and inflammatory arthropathies (including psoriatic arthritis, ankylosing spondylitis, systemic sclerosis, myositis, and other connective tissue diseases), were rigorously excluded on the basis of expert rheumatological evaluation.

Furthermore, RA_ane was strictly defined according to the Chinese diagnostic criteria for anemia, with HB levels of <120 g/l for adult males and < 110 g/l for adult nonpregnant females [33]. Patients receiving active, high-dose intravenous or oral iron therapy or erythropoiesis-stimulating agents within 2 weeks prior to sampling were explicitly excluded to minimize immediate therapeutic confounding. Strict baseline parity between the RA and RA_ane multiomics subcohorts was enforced to structurally control for potential demographic and clinical confounders prior to molecular analyses. Consequently, no statistically significant intergroup differences were observed regarding age, gender, disease duration, or concurrent therapeutic regimens (all *P* > 0.05; Table S2), establishing a highly balanced epidemiological foundation. Fasting plasma and fecal samples, alongside general clinical metadata and routine laboratory test results, were collected from all participants on the second day postadmission. The study protocol was approved by the Medical Ethics Review Committee of Dazhou Central Hospital, adhering to the Declaration of Helsinki, and all individuals provided written informed consent.

### 16S ribosomal RNA amplicon sequencing of human fecal samples

Fecal samples of the individuals were extracted using the E.Z.N.A. Soil DNA Kit (Omega Bio-tek, Norcross, GA, USA) for total DNA. After DNA testing, The V3 to V4 variable region of the 16*S* rRNA gene was amplified by polymerase chain reaction (PCR) using 338F (5′-actCCtacGGgaggCAGCAGG-3′) and 806R (5′-GGACTACHVGGGTWTCTAAT-3′). Finally, new amplicon sequence variants were extracted and analyzed according to the minimum sample sequence number. The analysis was done by the biotechnology company (Magi Bio, China) platform. Detailed methodology can be found in Supplementary Materials 1.

### Nontargeted metabolome detection of human plasma samples

Peripheral venous blood plasma was collected from the study individuals and analyze during liquid chromatography–tandem mass spectrometry (LC–MS/MS). Chromatographic separation was performed on an ACQUITY UPLC HSS T3 column (100 mm × 2.1 mm; internal diameter, 1.8 μm; Waters, USA) at 40 °C with a 2-μl injection volume. The binary mobile phase consisted of phase A (95% water, 5% acetonitrile, and 0.1% formic acid) and phase B (47.5% acetonitrile, 47.5% isopropanol, 5% water, and 0.1% formic acid). Raw LC–MS files were processed using Progenesis QI software (Waters) for peak alignment and aggregation. Batch variations were computationally mitigated through merged library searching and global retention time alignment. Features with a relative standard deviation of >30% in quality control samples were discarded. For metabolite annotation, MS1 mass tolerance was set to <10 parts per million, and metabolites were identified at Metabolomics Standards Initiative levels 1 and 2 using in-house libraries, Metlin, and LipidBlast. Data preprocessing included removal of metabolic features with >20% missing values, imputation of remaining missing values with the minimum value, sum normalization, and log_10_ transformation. Differential metabolites were selected on the basis of an orthogonal projections to latent structures–discriminant analysis variable importance in projection of >1.0 and nominal *P* < 0.05. Analyses were performed using the Biotech (Magi Bio, China) platform. Detailed methods can be found in the Supplementary Materials 1.

### RNA sequencing transcriptomics

Peripheral venous blood samples of the study individuals were extracted and detected by Agilent 2100 bioanalyzer (Agilent Technologies, CA, USA) and AMPure XP beads purified PCR products, and libraries were constructed. The NEBNext Ultra RNA Library Prep Kit for Illumina was used to perform library quality checks, then Illumina NovaSeq 6000 was used for synthesis and sequencing, and 150-bp paired-end readings were produced. Finally, the sequences were aligned to the reference genome using HISAT2 (v2.0.5). The analysis was done by the biotechnology company (Norogene, Beijing) platform. Detailed methodology can be found in Supplementary Materials 1.

### Four-dimensional data-independent acquisition quantitative proteome and phosphorylation-modified proteome analysis

Total proteins were extracted from peripheral venous blood samples. Protein quality was assessed using the Bradford assay, followed by proteolytic digestion. Data-dependent acquisition spectral libraries were constructed, and subsequent data-independent acquisition mode LC–MS analysis was performed. Retention time alignment was fully automated using the indexed retention time standard system within Spectronaut. Missing values for low-abundance proteins were retained as nulls without imputation to avoid artificial quantitative noise. Peptide–spectrum match and protein identification false discovery rates (FDRs) were strictly controlled at ≤1%. For differential expression screening, we applied a threshold of |fold change| ≥ 1.2 and nominal *P* < 0.05. This empirically established threshold for untargeted plasma proteomics was deliberately selected to minimize false-negative rates and ensure the retention of biologically critical, low-abundance regulatory proteins that are typically obscured by the massive dynamic range of the plasma proteome [34,35]. For the phosphorylation-modified quantitative proteome analysis, phosphorylated peptides were enriched using lyophilized binding buffer prior to data-dependent acquisition spectral library construction, while other procedures remained consistent with the standard proteomic workflow. All experimental data were analyzed by the biotechnology platform of Norogene (Beijing, China). Detailed methodologies are provided in Supplementary Materials 1.

### Quantitative PCR validation

The expression of the key gene 5-aminolevulinate synthase 2 (*ALAS2*) was validated by quantitative real-time PCR in independent samples. Detailed information regarding the study cohorts, RNA extraction, reverse transcription, primer sequences, and the quantitative real-time PCR protocol is provided in Supplementary Materials 1.

### Machine learning modeling and biomarker consensus

Rather than deploying a pure deep learning integrator, we conceptualized our architecture as a hybrid attention-based feature extractor, specifically designed to decode complex omics relationships while structurally preventing algorithmic overfitting in a mid-scale clinical cohort. Information leakage was strictly prevented by evaluating the unfiltered feature sets through a confined 5-fold stratified cross-validation pipeline across both omics modalities. Within each cross-validation fold, data scaling and principal components analysis (PCA) (reducing the feature space to 10 and 30 components for the metabolomic and transcriptomic datasets, respectively) were fitted exclusively on the training subset and subsequently applied to the validation subset. In this framework, PCA acts as a critical regularizer against the curse of dimensionality, while the core 3-layer transformer encoder decodes the complex, nonlinear dependencies among these principal components. The multihead self-attention mechanism was tailored to accommodate the distinct dimensionalities of the datasets, configuring 2 heads for the metabolomic data and 6 heads for the transcriptomic data. Substantial regularization was enforced using a dropout rate of 0.5 and an L2 weight decay penalty (*λ* = 0.1). The networks were optimized utilizing the Adam optimizer to minimize the binary cross-entropy loss, applying modality-specific learning rates (0.0005 for metabolomics and 0.0001 for transcriptomics) and early stopping patience thresholds (8 and 10 epochs, respectively).

A dual-filter strategy, integrating statistical significance with algorithmic consensus, was implemented to robustly guard against false positives and identify highly stable biomarkers. Specifically for the metabolomic data, we utilized a composite statistical threshold of variable importance in projection of >1.0 and a nominal *P* < 0.05. Strict FDR correction in highly collinear metabolic datasets frequently incurs excessive false negatives; therefore, we deliberately bypassed strict FDR. Instead, false-positive risks were mitigated by post hoc intersecting these classical differential features with the core biological drivers highlighted by the transformer’s self-attention weights. The analytical utility of the transformer architecture was rigorously justified by benchmarking its predictive performance against an ensemble of 5 stringently regularized traditional machine learning models (*K*-nearest neighbor [KNN], support vector machine [SVM], RF, recursive feature elimination-RF [RFE-RF], and XGBoost). Furthermore, rather than relying solely on deep learning metrics, this traditional ensemble was simultaneously deployed to serve as a downstream algorithmic consensus filter, ensuring that the finalized biomarkers were universally stable and algorithm-agnostic. Model performance was comprehensively evaluated using a pooled metric array comprising the area under the receiver operating characteristic (ROC) curve (AUC), accuracy, precision, recall, and F1-score. Precision–recall and calibration curves (computed using 5 quantile bins) were generated to rigorously validate the models’ positive predictive reliability and probability calibration. Detailed architectural configurations, benchmarking results, and feature selection methodologies are provided in Supplementary Materials 1*.*

### Statistical analyses

Anemia incidence was calculated using the initial hospital visit for diagnostic confirmation as the enrollment baseline time point for both RA and non-RA populations. Patients were categorized into annual cohorts (2020–2025) based on their year of hospital visit. The annual anemia incidence was calculated by dividing the number of newly diagnosed anemia cases in that year by the total number of individuals tested during that year. The 95% confidence intervals were computed using the Wilson score method. Differences in anemia rates between RA and non-RA groups within the same year were assessed using Pearson’s chi-square test, with statistical significance defined as *α* < 0.05 (2-tailed). A post hoc statistical power analysis was executed to verify the robustness of the multiomics discovery phase against potential sample size limitations. Furthermore, the independent associations between inflammatory markers and HB levels were evaluated using multivariate linear regression, strictly adjusting for clinical covariates including age, gender, disease duration, and treatment regimen. The analytical reliability of these associations was orthogonally validated across 3 distinct modeling approaches: standard linear regression, ridge regression, and RF. Multiple testing corrections were applied using the Bonferroni method. Downstream differential metabolite analysis and bacterial microbiome profiling were conducted via the Majorbio Cloud Platform (www.majorbio.com). This comprehensive pipeline encompassed α- and β-diversity evaluations, Kyoto Encyclopedia of Genes and Genomes (KEGG) pathway enrichment and topology analyses (kegg_v94.2), gene set enrichment analysis, and functional pathway inferences using Phylogenetic Investigation of Communities by Reconstruction of Unobserved States (PICRUSt2, v2.2.0-b). Statistical analyses and data visualization were performed using GraphPad Prism (v8.0) and SPSS Statistics (v25.0.0.0). Functional enrichment analysis and protein–protein interaction networks were conducted with STRING (https://string-db.org) and visualized in Cytoscape (v3.7.2). General bioinformatics and statistical computing were carried out in R (v4.4.2) through RStudio. The core deep learning modeling, including the implementation of the transformer architecture, was conducted in Python (v3.10.10). The models were built and trained using the PyTorch framework (v2.6.0) with the following key libraries: scikit-learn (v1.6.1) for data preprocessing, feature selection, and traditional machine learning models (KNN, SVM, RF, and XGBoost); pandas (v2.2.3) and NumPy (v2.0.2) for data manipulation; and Matplotlib (v3.10.1) and Seaborn (v0.13.2) for visualization.

## Results

### Baseline characteristics of the cohorts and multiomics population

**Fig. 2.**
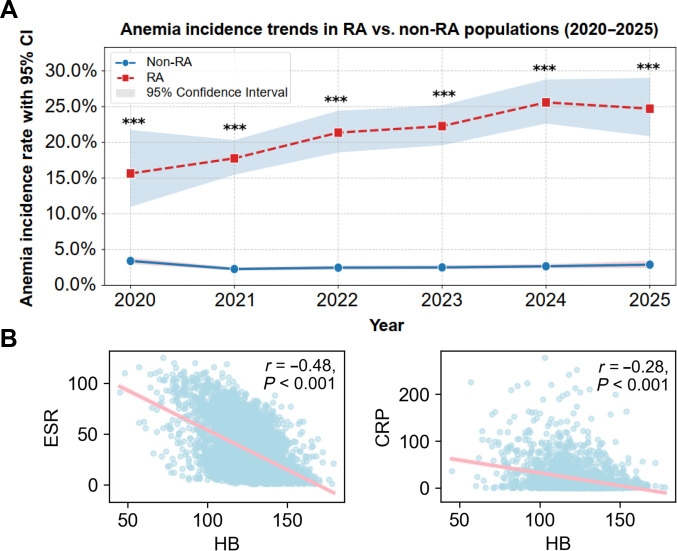
Clinical characterization of rheumatoid arthritis (RA)-complicated anemia. (A) Line graph of annual anemia incidence between the RA and non-RA cohorts from 2020 to 2025. Red, the RA cohort; blue, the non-RA cohort. Differences in anemia incidence between the 2 cohorts are indicated by significance markers. (B) Correlation analysis between hemoglobin (HB), C-reactive protein (CRP), and erythrocyte sedimentation rate (ESR) in the RA cohort. ***P < 0.001.

The analytic workflow is shown in Fig. 1. The cohort included 4,114 patients with RA (77.9% female; mean age: 55.8 ± 12.6 years) and 100,934 non-RA individuals (46.3% female; mean age: 41.8 ± 16.3 years) (Table S1). A post hoc power analysis confirmed that the multiomics sample size retained adequate statistical power (97.9%) to detect moderate molecular differences. Crude incidence rates of anemia by onset year are illustrated in Fig. 2A. In the RA cohort, HB levels negatively correlated with C-reactive protein (CRP) and erythrocyte sedimentation rate (ESR) (Fig. 2B). Participants underwent sample collection, sequencing, characterization, and functional analysis, followed by aggregated multiomics analysis. Clinical characteristics (age, sex, HB, CRP, RF, and ESR) are provided in Table S2. ESR, CRP, and IL-6 levels were significantly higher in patients with RA_ane compared to the RA group (*P* < 0.001), with similar trends in SLE and gout (Table S3). In addition, clinical data revealed a significant correlation between HB levels and the expression of CRP, ESR, IL-6, and the disease activity score of 28 joints (DAS28). However, no linear regression correlation was observed between RF and HB levels (Fig. S1A to E).

### Identification of key RA_ane biomarkers by aggregating differential analysis and machine learning

**Fig. 3.**
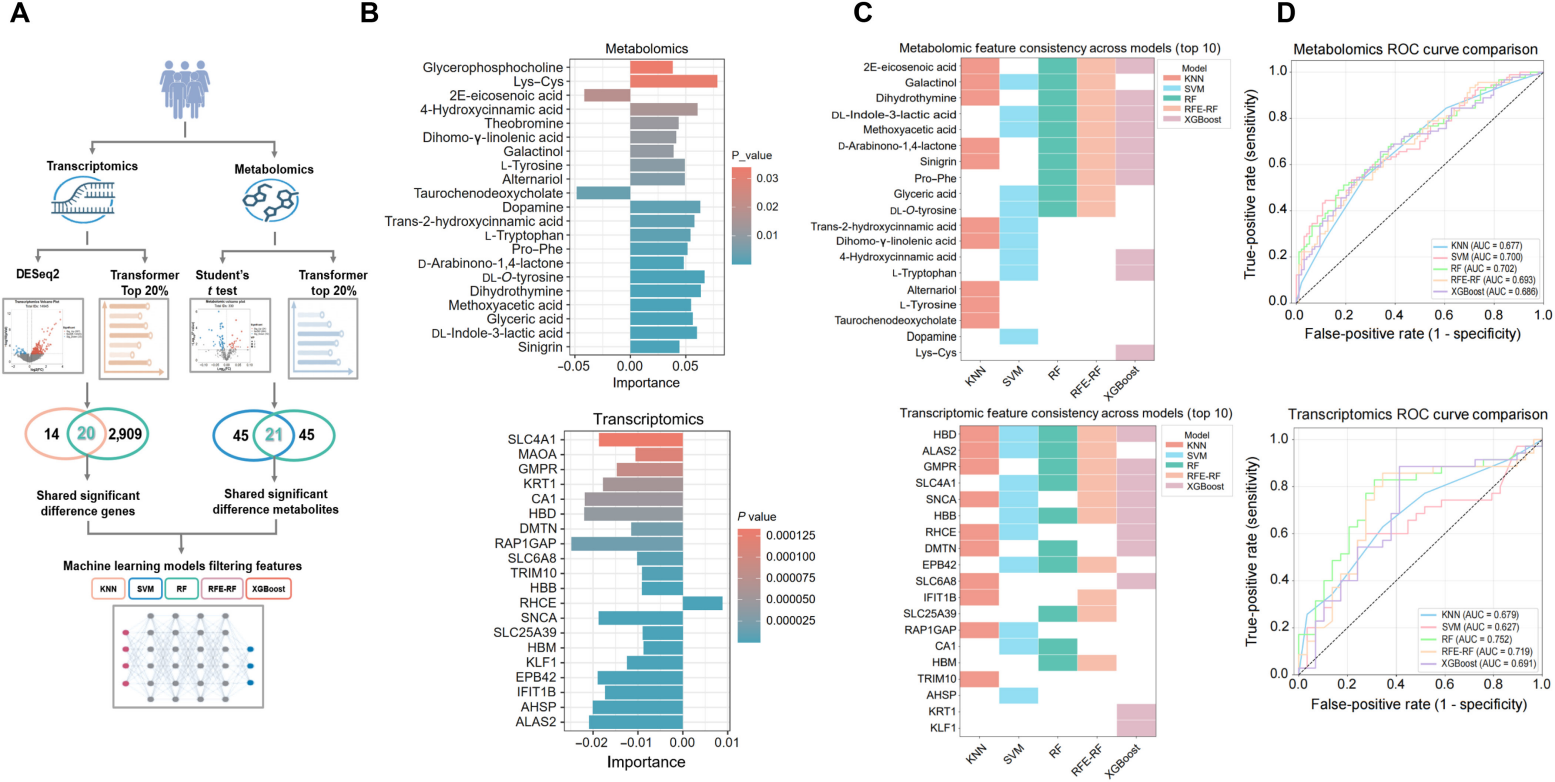
Screening of important features using differential analysis and machine learning models. (A) Flowchart illustrating the analysis pipeline based on metabolomic and transcriptomic differential analysis combined with machine learning models. (B) Common features screened by differential analysis and by transformer architecture were ranked on the basis of the weights, and the top features ranked by their importance are shown. The importance values of the features are labeled at the bottom, where red represents high *P* value and blue represents low *P* value. (C) All common features identified through variance analysis and transformer architecture were further screened using 5 machine learning models. The top 10 ranked features obtained for each model were concatenated and are shown as model-specific patterns. Among these, 19 metabolomic and 19 transcriptomic features were screened by all 5 models. (D) Receiver operating characteristic (ROC) curves of 5 machine learning models in metabolomic and transcriptomic datasets. RA, rheumatoid arthritis; RA_ane, rheumatoid arthritis complicated by anemia; KNN, *K*-nearest neighbors; SVM, support vector machine; RF, random forest; RFE-RF, recursive feature elimination-RF; XGBoost, extreme gradient boosting.

To identify abnormal metabolic and transcriptional profiles in the RA_ane population, we conducted untargeted metabolomic profiling on fasting peripheral venous blood plasma and transcriptome sequencing on peripheral blood mononuclear cells (PBMCs). We initially screened for differential features using a transformer-based architecture combined with variance analysis. key features were subsequently refined through an algorithmic consensus of 5 conventional machine learning models (Fig. 3A). Our analysis identified 66 differential metabolites between RA_ane and RA populations, as determined by Student’s *t* test (Fig. S2A). Furthermore, preprocessing of the transcriptomic expression matrix (by excluding rows with more than 50% zero values and normalizing the data using the DESeq2 package) yielded a total of 14,645 genes. We identified 34 differentially expressed genes (DEGs) in RA_ane compared to RA, comprising 33 up-regulated and 1 down-regulated genes (FDR < 0.01, |fold change| > 2) (Fig. S2B).

Subsequently, we analyzed the weight scores for all the differentially abundant metabolites and genes between the 2 groups using the transformer architecture. The metabolomic analysis extracted features ranked in the top 20% by importance and intersected them with the classically defined differential metabolites, yielding 21 statistically significantly altered metabolites. Similarly, the transcriptomic analysis identified genes ranked in the top 20% by importance, yielding 20 genes consistent with the classical DEGs. The top 20 ranked features based on their importance scores, along with their corresponding *P* values, are presented (Fig. 3B and Fig. S2C to F). To establish a robust set of biomarkers for further investigation, we subsequently aggregated the top 10 features from each of the 5 models through a consistency analysis (Fig. 3C). The stability of these features was highlighted in attention-based importance heatmaps, which identified l-tryptophan and glyceric acid among the top 20 metabolic features and *ALAS2* and carbonic anhydrase 1 (*CA1*) among the top 30 genes. Furthermore, the ROC curves generated using these models demonstrated strong predictive performance (Fig. 3D, Figs. S3A to F and S4A to F, and Table S4). Aggregating multiomics differential analysis with transformer architecture and machine learning models substantially enhanced the identification of core features associated with RA_ane.

### Multiomics functional profiling reveals the central role of glycine, serine, and threonine metabolism pathway in RA_ane pathogenesis

**Fig. 4.**
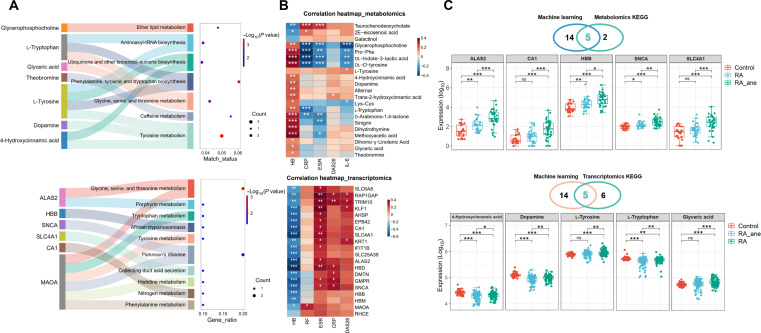
Functional analysis of metabolomics and transcriptomics revealed the importance of the glycine, serine, and threonine metabolism (GSTM) pathway in rheumatoid arthritis complicated by anemia (RA_ane). (A) Kyoto Encyclopedia of Genes and Genomes (KEGG) analysis was carried out for the common differential metabolites and differential genes screened by differential analysis and transformer, where the same pathways were marked in red text. (B) Correlation analysis of 21 shared differential metabolites and 20 shared differential genes with hemoglobin (HB), C-reactive protein (CRP), erythrocyte sedimentation rate (ESR), interleukin-6 (IL-6), and disease activity score of 28 joints (DAS28), where blue color represents negative correlation and red color represents positive correlation. (C) Among all the differential features screened by the 5 machine learning models, 5 metabolites, and 5 genes overlapped with the features on the KEGG pathway, and the box plots of their expression levels in the control, RA, and RA_ane groups are presented. RF, rheumatoid factor. *P < 0.05, **P < 0.01, and ***P < 0.001. ns, not significant.

We performed KEGG topology analysis on the 21 key metabolites identified, revealing that the glycine, serine, and threonine metabolism (GSTM) pathway, along with tyrosine metabolism and glyoxylate and dicarboxylic acid metabolism, exhibited the highest impact values (Fig. S5A). Notably, glyceric acid and l-tryptophan were enriched in the GSTM and glyoxylate and dicarboxylic acid metabolism pathways, whereas l-tyrosine, dopamine, and 4-hydroxycinnamic acid were enriched in the tyrosine metabolic pathway (Fig. 4A). Further analysis of the transcriptomic data revealed significant perturbations in the GSTM pathway, with genes such as *ALAS2* and monoamine oxidase A (*MAOA*) showing enrichment (Fig. 4A and Fig. S5B). Gene set enrichment analysis also indicated dysregulation of GSTM pathway, as well as in porphyrin and chlorophyll metabolism (Fig. S5C).

Correlation analysis between the key intersecting biomarkers and clinical inflammatory markers in RA demonstrated that most of key metabolites and genes were significantly associated with HB, CRP, and ESR. Specifically, metabolites such as glyceric acid and l-tryptophan showed significant positive correlations with HB, whereas l-tryptophan exhibited a significant negative correlation with CRP. In addition, *ALAS2* and *CA1* showed a significant negative correlation with HB but a significant positive correlation with ESR (Fig. 4B). Based on the algorithmic consensus and KEGG screening, we finalized 5 key differential metabolites and 5 DEGs. The relative abundances of glyceric acid, l-tryptophan, 4-hydroxycinnamic acid, dopamine, and l-tyrosine were significantly decreased in RA_ane, whereas the expression levels of *ALAS2*, *CA1*, HB subunit β (*HBB*), synuclein-α (*SNCA*), and solute carrier family 4 member 1 (*SLC4A1*) were robustly up-regulated (Fig. 4C). Functional analyses of the multiomics data suggest that altered profiles of glyceric acid, l-tryptophan, and *ALAS2* are prominent molecular hallmarks in the RA_ane population. Furthermore, these aggregated findings suggest that the GSTM pathway is fundamentally implicated in the pathophysiology of anemia.

### Multidimensional functional validation and comparative disease analysis underscore the importance and uniqueness of GSTM in RA_ane

**Fig. 5.**
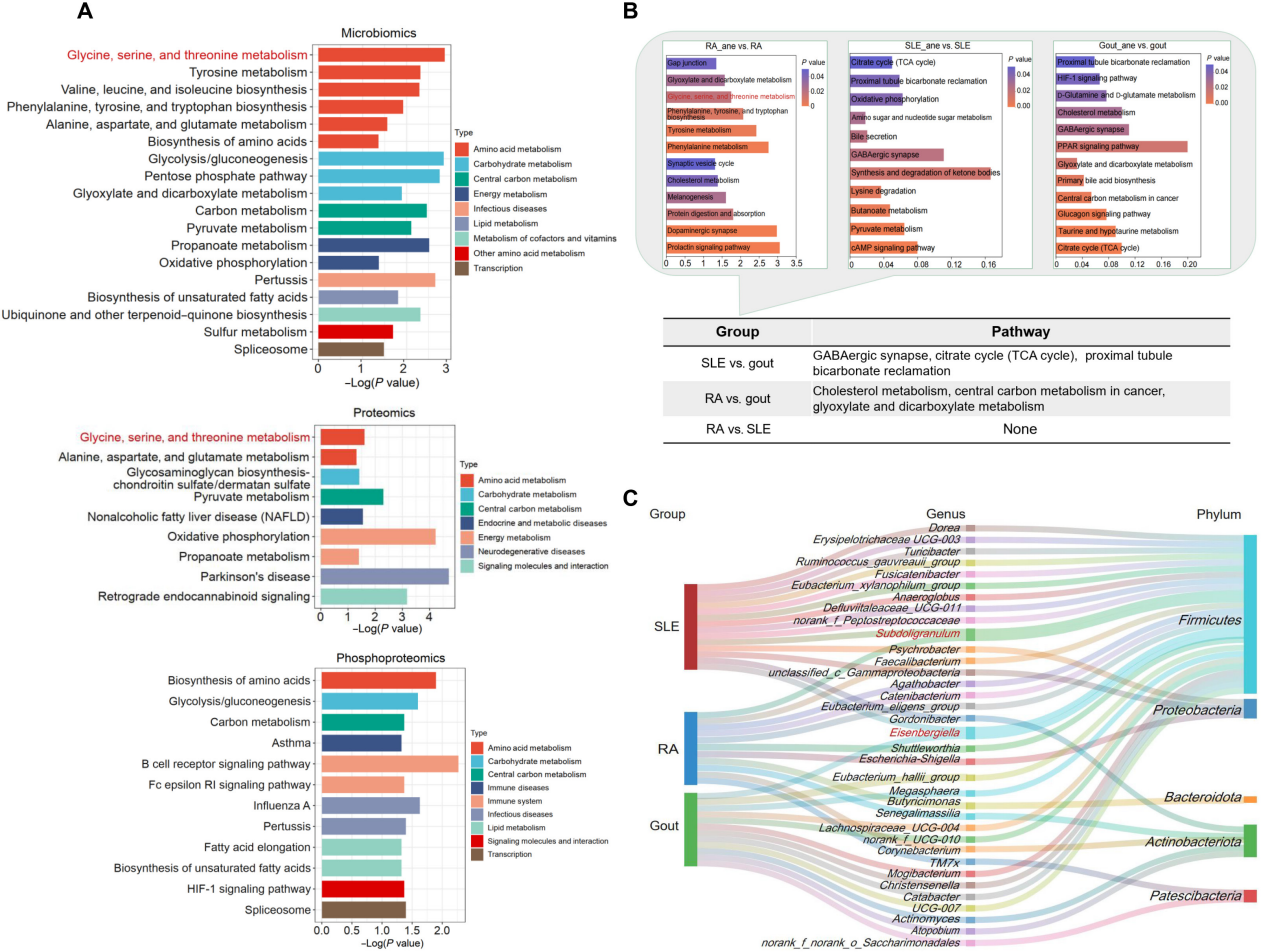
Validation of the importance of glycine, serine, and threonine metabolism (GSTM) from multiomics data and disease comparison analysis. (A) Functional prediction analysis of microbiomics and Kyoto Encyclopedia of Genes and Genomes (KEGG) enrichment analysis of proteomics and phosphoproteomics were performed. (B) Metabolomic analysis was performed for rheumatoid arthritis (RA), systemic lupus erythematosus (SLE), and gout anemia populations. The top half of the figure shows the results of KEGG enrichment analysis of the differential metabolites, and the bottom half of the figure presents the same pathways in the comparison of SLE with gout, RA with gout, and RA with SLE. The top half shows the KEGG enrichment analysis of differential metabolites, and the bottom half presents the same pathways in the comparisons of SLE with gout, RA with gout, and RA with SLE. (C) Microbiomic analysis of RA, SLE, and gout anemia populations, with statistics of differential microorganisms at the genus level and the phylum level, and the same genera are labeled in red font. RA_ane, rheumatoid arthritis complicated by anemia; SLE_ane, SLE complicated by anemia; gout, gouty arthritis; gout_ane, gout complicated by anemia. TCA, tricarboxylic acid cycle; GABAergic, gamma-aminobutyric acidergic; cAMP, cyclic adenosine monophosphate; PPAR, peroxisome-proliferator-activated receptor.

To further examine the complex interactions between gut microbiota dysregulation and disease, we performed 16*S* rRNA sequencing on fecal samples from individuals in the control, RA, and RA_ane groups. Taxonomic composition analysis revealed distinct dysbiotic signatures. Specifically, compared to the RA group, the RA_ane microbiome exhibited a significant expansion of the pathobiont *Escherichia–Shigella*, alongside a concurrent profound depletion of the immunoregulatory commensals *Faecalibacterium* and *Subdoligranulum* (Fig. S6A). Significant correlations were also observed between these differential microbial genera and clinical markers, including HB, DAS28, and other inflammatory indices (Fig. S6B). PICRUSt2 analyses revealed differences in the GSTM pathway between the RA_ane and RA groups and identified 5 genera that highly correlated with HB and the GSTM pathway (Fig. 5A and Fig. S6C and D). Given the limitations of 16*S* rRNA-based functional inference via PICRUSt2, these associated metabolic perturbations are interpreted strictly as hypothesis-generating exploratory associations (Fig. S6E to G). These results suggest that intestinal bacterial dysbiosis is intrinsically linked to RA_ane and associated with GSTM pathway disruption.

Subsequently, we utilized proteomic and phosphoproteomic data to conduct functional enrichment analysis of differentially phosphorylated proteins. The proteomic analysis revealed enrichment of the GSTM pathway, in which serine hydroxymethyltransferase 1 (SHMT1) and phosphoglycerate dehydrogenase (PHGDH) were identified as key differentially expressed proteins (Fig. 5A and Fig. S7A to C). Phosphoproteomic enrichment analysis revealed pathways related to hypoxia-inducible factor 1 (HIF-1) signaling, lipid metabolism, and heme binding (Fig. 5A and Fig. S7D). We also found that PHGDH and SHMT1 were negatively associated with HB (Fig. S7E). Protein–protein interaction analysis indicated that proteins encoded by *CA1* and *HBB* exhibited altered phosphorylation statuses in RA_ane (Fig. S7F). These results indicate that the levels of phosphorylated proteins encoded by *SHMT1*, *PHGDH*, and *CA1* were elevated in the RA_ane population, suggesting an increased susceptibility to alterations in the GSTM and HIF-1 signaling pathways.

To corroborate the specificity of the GSTM pathway in RA_ane, we performed a comparative disease analysis using SLE and gout cohorts. Volcano plots illustrated distinct plasma metabolite profiles between anemic and nonanemic patients (Fig. S8A and B). Pathway enrichment analyses of differential metabolites across the RA, SLE, and gout populations indicated no overlapping pathways between the RA-anemic and SLE-anemic groups. However, 3 common pathways were identified in the gout-anemic population, including cholesterol metabolism, central carbon metabolism in cancer, and glyoxylate and dicarboxylate metabolism (Fig. 5B). Microbiome analyses using PICRUSt2 did not show enrichment of GSTM pathway in patients with SLE and gout. Despite the autoimmune nature shared among these diseases, the gut microbiota of anemic patients was highly disease specific, with unique dominant genera observed in each group (Fig. 5C and Fig. S8C to F). Thus, our findings suggest that the GSTM pathway dysregulation is specific to the RA_ane process, highlighting its potential mechanistic and diagnostic significance.

### Clinical diagnostic model development and GSTM-based multidimensional molecular mapping for RA_ane

**Fig. 6.**
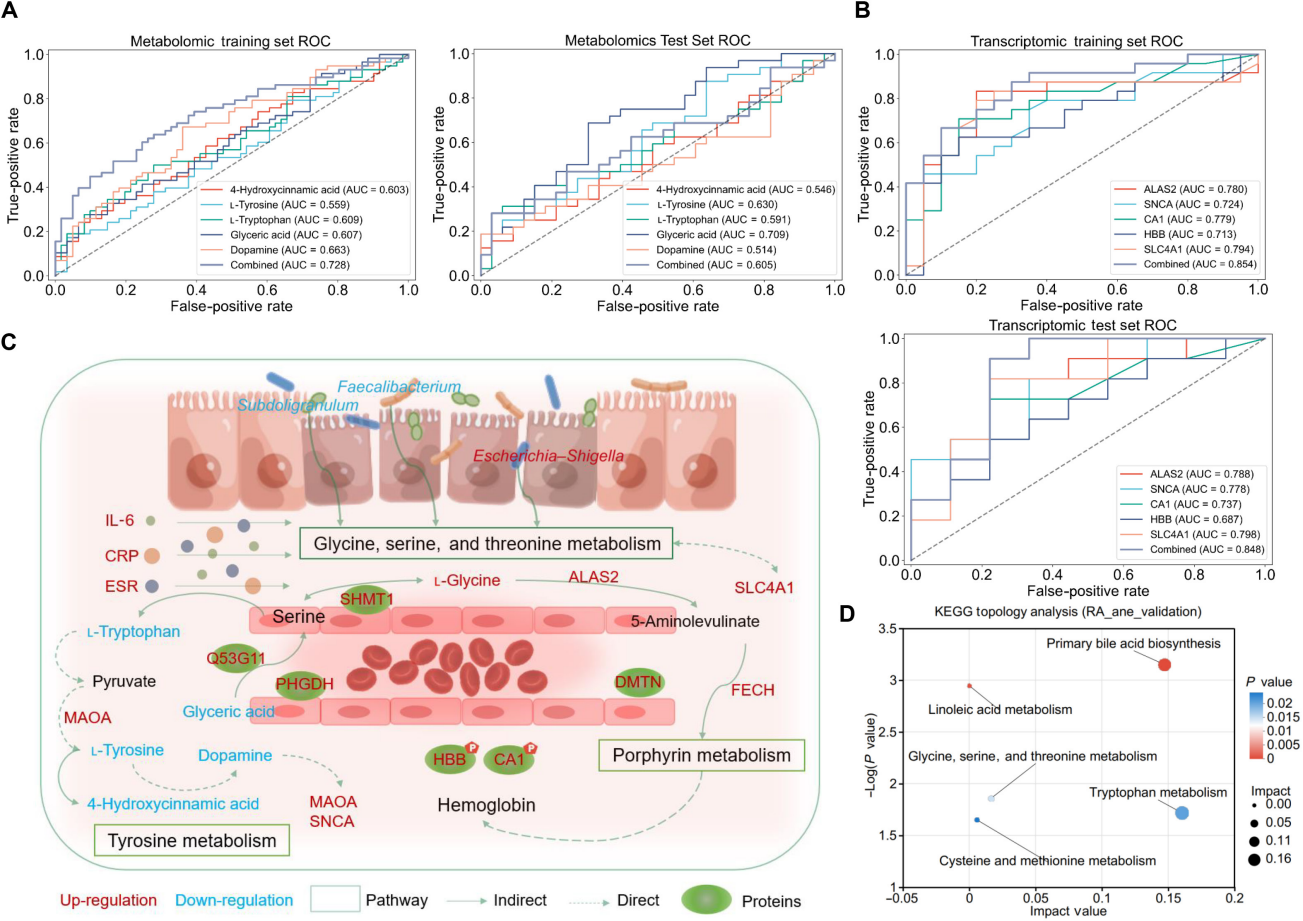
Diagnostic modeling and molecular mechanism analysis. (A) Receiver operating characteristic (ROC) curves of the metabolomic-based diagnostic model, constructed using glyceric acid, l-tryptophan, 4-hydroxycinnamic acid, dopamine, and l-tyrosine in the training and testing cohorts. (B) ROC curves of the transcriptomic-based diagnostic model, built using *ALAS2*, *CA1*, *HBB*, *SNCA*, and *SLC4A1* in the training and testing cohorts. (C) This is a schematic diagram of the rheumatoid arthritis (RA) complicated by anemia (RA_ane) state mechanism hypothesis based on the glycine, serine, and threonine metabolism (GSTM) pathway. (D) Topological analysis of the Kyoto Encyclopedia of Genes and Genomes (KEGG) pathway enriched in the independent metabolomic validation cohort, underscoring the central role of the GSTM pathway. SLE, systemic lupus erythematosus; SLE_ane, SLE complicated by anemia; gout, gouty arthritis; gout_ane, gout complicated by anemia.

Based on the multiomics differential analyses, machine learning screening, and functional enrichment results, we selected 5 core metabolites and 5 genes to construct diagnostic classifiers. Patients with RA were partitioned into training and internal test sets at a 7:3 ratio. The diagnostic performance was excellent, achieving AUC values of 0.728 (training) and 0.605 (testing) for the metabolomic model and 0.854 (training) and 0.848 (testing) for the transcriptomic model (Fig. 6A and B). Furthermore, to rigorously evaluate the models’ reliability beyond standard ROC metrics, we generated precision–recall and calibration curves. Both the transcriptomic (average precision [AP] = 0.74) and metabolomic (AP = 0.76) models demonstrated high positive predictive precision and excellent probability calibration, confirming their robustness in clinical risk stratification (Fig. S9). A combined analysis of the metabolomic and transcriptomic data confirmed that the GSTM pathway was significantly enriched (Fig. S10). We further aggregated key differential plasma metabolites, gut microbiota, DEGs, differential proteins, and phosphorylated proteins to construct a multidimensional molecular map of RA_ane centered on the GSTM pathway, which indirectly associates HB levels alongside pathways such as porphyrin metabolism (Fig. 6C).

To further strengthen the robustness of our findings, we successfully validated the key metabolic discoveries related to the l-tryptophan and the GSTM pathway in an independent external cohort (22 RA_ane versus 65 RA individuals) (Fig. 6D and Fig. S11A to C). Moreover, quantitative PCR (qPCR) analysis confirmed the significant up-regulation of a pivotal GSTM pathway gene, *ALAS2*, in the RA_ane group compared to RA group (*P* = 0.038, *n* = 8 per group; Fig. S11D). This provides direct, orthogonal experimental support for the dysregulation of this transcript in RA-related anemia.

### Multivariate association analysis of inflammation and anemia

To quantitatively assess the residual impact of clinical confounders on HB variance, we constructed a comprehensive multivariate framework (Table S5). While physiological covariates such as age and gender exhibited minor baseline effects (feature importance = 0.058), their explanatory power was vastly overshadowed by systemic inflammation. Notably, ESR emerged as the overwhelmingly dominant predictor of anemia (feature importance = 0.640, *P* < 0.001), with a negative regression coefficient (−0.383). Concurrent therapeutic regimens exhibited negligible immediate impact (feature importance = 0.0048). This supports that our omics signatures capture the authentic pathophysiology of RA_ane independent of major demographic imbalances.

## Discussion

In this study, we identified distinct differential features between RA and RA_ane using a transformer-based architecture alongside multiomics aggregation, followed by the application of 5 conventional machine learning algorithms as an algorithmic consensus filter. Functional enrichment analysis revealed significant alterations in the GSTM pathway specifically in RA_ane. Disruption of this pathway was substantiated at both microbial and protein levels. Notably, comparative analyses with anemic patients from SLE and gout cohorts demonstrated unique metabolic and microbiomic patterns, highlighting the specificity of GSTM pathway alterations in RA_ane. We developed classification models based on 5 key metabolites and 5 genes, which exhibited excellent predictive performance across both training and test sets. These findings underscore the pivotal role of GSTM pathway in rheumatoid anemia, highlighting its potential to inform future clinical diagnostics and mechanism-driven research.

Previous studies have linked the GSTM axis to various critical molecular processes [36]. From a pathophysiological perspective, we propose a mechanistic cascade driven by inflammatory metabolic hijacking [37]. Chronic systemic inflammation in RA induces substantial oxidative stress, prompting highly activated immune cells to heavily consume serine and glycine for glutathione synthesis as an antioxidant defense. This systemic metabolic shift profoundly depletes circulating glycine, an obligate substrate for heme biosynthesis [38,39]. Furthermore, the significant reduction in glyceric acid observed in our cohort reflects this severely disrupted serine/glycine axis within the GSTM pathway, which restricts the one-carbon metabolism critically required for nucleotide biosynthesis during rapid erythroblast proliferation [40]. Concurrently, the pronounced depletion of l-tryptophan is a classic hallmark of chronic inflammation. Proinflammatory cytokines, particularly TNF-α and interferon-γ, potently induce indoleamine 2,3-dioxygenase, rapidly shunting l-tryptophan into the kynurenine pathway. This process not only deprives rapidly dividing erythroid progenitors of an essential amino acid but also generates downstream kynurenine metabolites that directly suppress erythropoiesis [41,42]. Ultimately, this combination of toxic amino acid starvation and impaired nucleotide synthesis creates a highly hostile environment for erythropoiesis.

Furthermore, our multiomics and qPCR validations consistently revealed a significant up-regulation of ALAS2, the rate-limiting enzyme in heme biosynthesis. Rather than indicating successful heme production, this paradoxical elevation reflects a robust but futile compensatory response to this profound metabolic stress and systemic iron restriction characteristic of ACD. Elevated IL-6 induces hepcidin, driving functional intracellular iron deficiency and iron sequestration [43,44]. Faced with this resulting heme deficiency and tissue hypoxia, erythroid progenitors mount a compensatory transcriptional up-regulation of ALAS2. In addition, severe anemic stress frequently triggers the premature release of immature erythroid precursors into the peripheral circulation, heavily contributing to the elevated ALAS2 signature detected in our PBMC transcriptomics [5]. We therefore posit that the immunometabolic disruption in RA_ane arises from a synergistic clash: a proinflammatory cytokine milieu driving iron restriction and erythroid suppression, combined with intrinsic deficiencies in glycine–serine–threonine metabolism that collectively impair heme synthesis.

While our molecular profiling inherently provides a cross-sectional snapshot, we integrated comprehensive clinical metadata to bridge the conceptual gap regarding temporal causality. The anemia prevalence within our molecular subcohort closely mirrored the epidemiological baseline of our broader 2020–2025 longitudinal clinical cohort, confirming its temporal representativeness. Furthermore, stratifying our metabolomic cohort by disease duration revealed a remarkably balanced distribution between patients with RA and RA_ane across early (≤2 years), intermediate (2 to 10 years), and long-standing (>10 years) disease stages. This uniform clinical distribution provides compelling evidence that GSTM dysregulation is not merely a terminal consequence of prolonged structural damage but rather an early and persistent metabolic shift that occurs in parallel with the onset of inflammation.

In our exploratory microbiome analysis, the RA_ane cohort was characterized by a distinct dysbiotic shift, notably the profound depletion of the immunoregulatory commensal *Faecalibacterium* and the concurrent expansion of the pathobiont *Escherichia–Shigella.* This aligns with previous research demonstrating that *Faecalibacterium* is reduced in individuals with iron deficiency anemia and recovers following iron supplementation [45]. Simultaneously, lipopolysaccharide, a primary component of the *Escherichia*–*Shigella* cell wall, is strongly associated with increased intestinal mucosal permeability and exacerbated systemic inflammatory responses [19,46]. Crucially, because our microbiome functional profiles were indirectly inferred via 16*S* rRNA sequencing utilizing PICRUSt2, these metabolic pathway predictions are inherently limited. Consequently, we interpret this specific dysbiotic shift strictly as an inflammatory correlate rather than a definitive, causal driver of the systemic GSTM pathway dysregulation. Nevertheless, this microbial disturbance suggests that patients with RA_ane endure a synergistic burden of mucosal inflammation and iron dysregulation, which collectively is associated with clinical severity.

Methodologically, rather than deploying a pure deep learning integrator, we conceptualized a hybrid PCA–transformer architecture to successfully decipher complex omics associations. Within this framework, PCA acts as a critical early regularizer to mitigate the “curse of dimensionality” inherent in mid-scale clinical cohorts, enabling the transformer to effectively decode the complex, nonlinear dependencies among the condensed principal components. The predictive superiority of this hybrid architecture was rigorously benchmarked against conventional machine learning models, demonstrating its robust diagnostic capabilities. Notably, we observed a discrepancy in diagnostic performance between the transcriptomic model (AUC: 0.854) and the metabolomic model (AUC: 0.728). This performance discrepancy indicates that PBMC transcriptomes capture stable, upstream immunologic drivers, whereas the plasma metabolome reflects dynamic, downstream physiological readouts that are inherently more susceptible to environmental perturbations and analytical noise [47,48]. Transcriptomic signatures derived from PBMCs represent a relatively stable, genetically anchored cellular state. They effectively capture the long-term immunologic conditioning induced by chronic RA inflammation. In contrast, the circulating plasma metabolome is highly dynamic. It is exquisitely sensitive to immediate environmental perturbations, including circadian rhythms, recent dietary intake, and transient microbiota fluctuations [48]. Furthermore, bootstrap statistical testing revealed no significant difference between the training and testing AUCs (95% confidence interval of difference: −0.038 to 0.298). This statistically confirms that the observed variance is not driven by algorithmic overfitting but is primarily attributable to the restricted sample size of our internal test cohort (*n* = 65; comprising 32 RA_ane and 33 RA individuals), making the evaluation metrics inherently sensitive to cohort heterogeneity. The internal stability of the model was further reinforced by a robust 5-fold cross-validation AUC of 0.658. Consequently, while the metabolic signature provides a critical real-time snapshot of the systemic biochemical phenotype, the PBMC transcriptome inherently yields a more sustained and robust diagnostic signal.

Importantly, while the transformer excels in overarching prediction to rigorously guard against false positives within these high-dimensional and noisy data spaces, we strategically deployed 5 conventional machine learning models (KNN, RF, RFE-RF, SVM, and XGBoost) in the downstream feature selection phase. Rather than serving solely as competing integrators, these algorithms functioned collectively as a stringent consensus filter. This ensemble approach effectively mitigates algorithm-specific biases, ensuring that our finalized biomarkers are universally stable and biologically reproducible [49]. Our multivariate analyses further substantiated the central role of systemic inflammation in the pathogenesis of RA anemia. ESR, emerging as the strongest independent predictor, directly supports the pathomechanism that chronic inflammation is fundamentally linked to both the onset and severity of anemia. Notably, while our multivariate models indicated that concurrent therapeutic regimens exerted minimal immediate effects on HB variance, we acknowledge that the cumulative impact and subclinical toxicities of historical treatments remain potential residual confounders.

Several limitations should be acknowledged. The retrospective and cross-sectional design precludes definitive causal inference, and we cannot fully exclude residual effects from historical iron supplementation or other unrecorded past treatments. The lack of comprehensive iron panels also means that the RA_ane group likely represents a mixed etiology, predominantly anemia of chronic disease (ACD). In addition, because the transcriptomic and metabolomic data came from largely nonoverlapping subcohorts, we could not construct a direct joint multiomics predictive model. Regarding generalizability, core metabolic signatures were validated in an independent external cohort (22 RA_ane and 65 RA patients), but the limited availability of perfectly matched multiomics public datasets restricts the scale of full validation. Therefore, these findings should be interpreted as robust proof-of-concept signatures that require confirmation in large-scale prospective studies.

## Conclusion

In summary, we used a machine learning framework based on the transformer architecture, combined with a multiomics strategy, to systematically analyze molecular interactions in RA_ane. This is the first report to demonstrate that the GSTM pathway indirectly regulates HB biosynthesis through porphyrin synthesis, thereby mediating RA_ane development. These findings highlight the core regulatory role of the GSTM pathway in RA_ane and suggest a potential molecular target for clinical intervention, offering a new theoretical basis for precise treatment of RA-related inflammation and anemia.

## Ethical Approval

This study was approved by the Medical Ethics Review Committee of Dazhou Central Hospital (ID numbers: 2021022, 2023040, and 2025159), and all the individuals signed informed consent.

## Data Availability

The metabolomic, microbiomic, transcriptomic, proteomic, and phosphoproteomic data are available from the corresponding author upon reasonable request. The custom code supporting the findings of this study is publicly available at GitHub: https://github.com/8426-ztc/RA.

## Supplementary Materials

Supplementary 1 Supplementary Materials 1 Supplementary Materials 2 Figs. S1 to S11 Tables S1 to S5
