# Supplementary material for: Transformer-Based Multiomics Study Identifies Important Role of Glycine, Serine, and Threonine Metabolism Pathway in Rheumatoid Arthritis Complicated by Anemia
Source: Comput Struct Biotechnol J. 2026 May 7;35(1):0075. doi: 10.34133/csbj.0075 (PMC13150071; doi:10.34133/csbj.0075)
Supplement: Supplementary 1 — Supplementary Materials 1 Supplementary Materials 2 Figs. S1 to S11 Tables S1 to S5 [file csbj.0075.f1.zip › Supplementary Figures and Tables.pdf]

Title: Transformer-based multi-omics study identifies important role of glycine, serine and threonine metabolism pathway in rheumatoid arthritis complicated by anemia

Authors: Jiaxin Huang[1*], Yuanli Wei[2*], Dongmei Wang[2*], Jianghua Chen[3], Congcong Jian[4], Xiaoting Zhu[4], Shilin Li[4], Jie Zhang[4], Tingting Wang[2], Caizhen Liu[2], Lingli Wei[2], Jing Gao[2], Jing Zhu[5], Qinghua Zou[6 #], Jianhong Wu[2 #], Fanxin Zeng[1,4,7#].

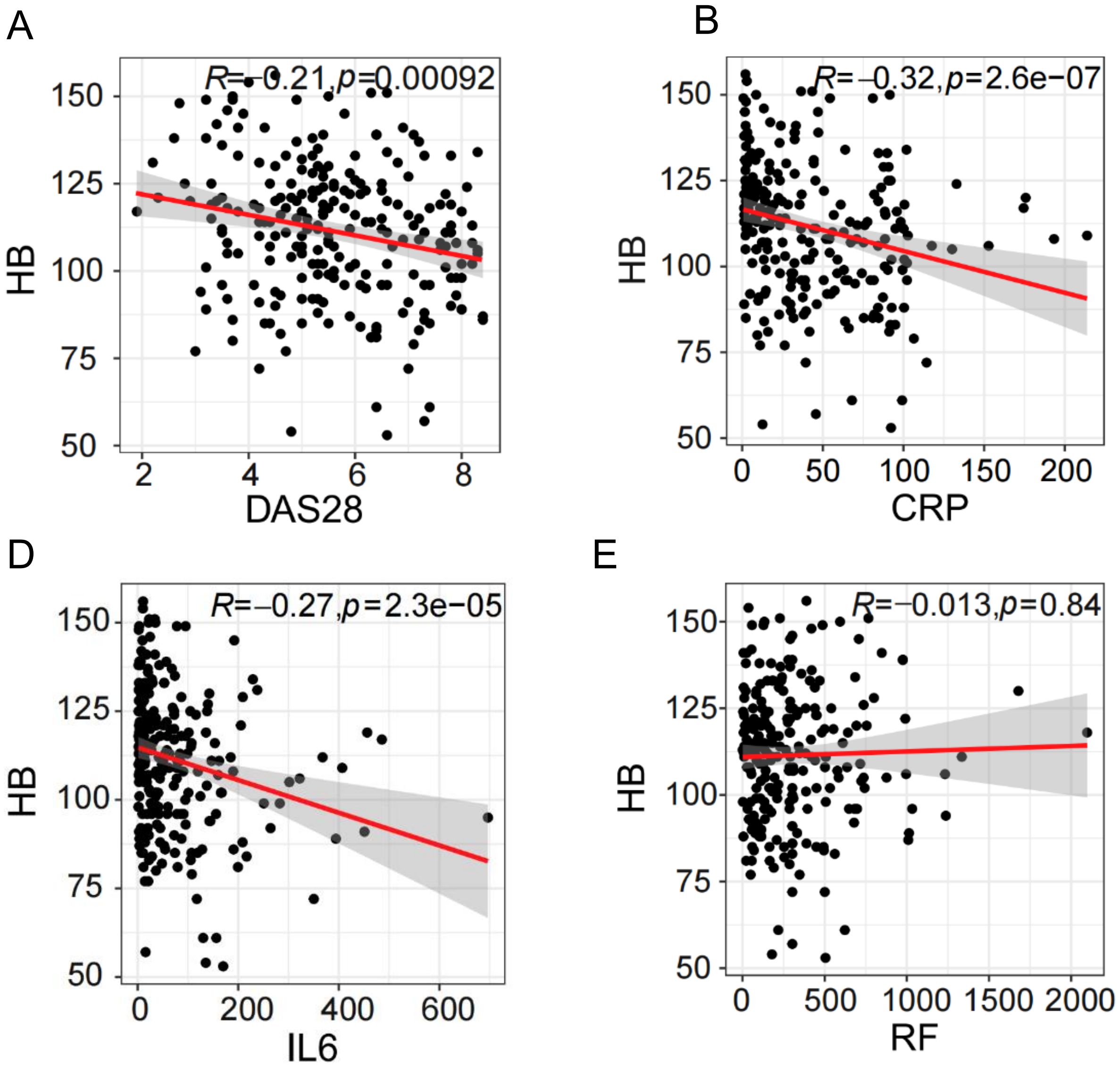

Supplementary Figure 1: Clinical data found a correlation between HB, inflammation levels, and disease activity. (A-E) Scatterplot of the spearman correlation of HB with DAS28, CRP, ESR, IL6 and RF, respectively. DAS28: disease activity score in 28 joints; CRP, C-reactive protein; ESR, erythrocyte sedimentation rate; IL6, interleukin 6; RF: rheumatoid factor.

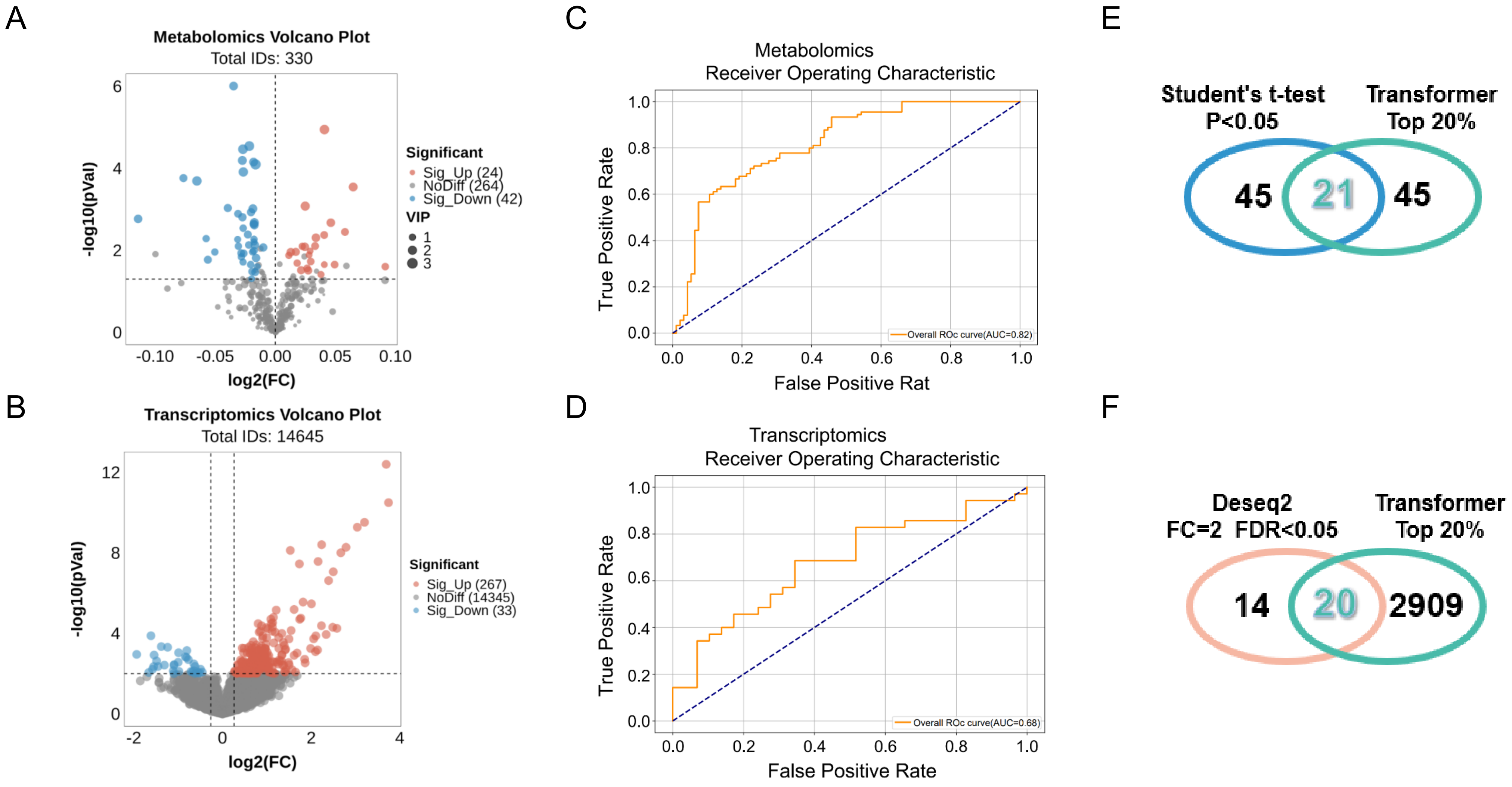

Supplementary Figure 2: Metabolomics and transcriptomics important features screening. (A) Metabolomics difference volcano map. (B) Transcriptomics difference volcano map. (C) All features of metabolomics are used in ROC curves obtained from transformer architecture. (D) All features of transcriptomics are used in ROC curves obtained from transformer architecture. (E) Metabolomics Student's t-test difference analysis with Venn diagram of the top 20% of transformer architecture importance. (F) Venn diagram of transcriptomics DESeq2 difference analysis with the top 10% of transformer architecture importance.

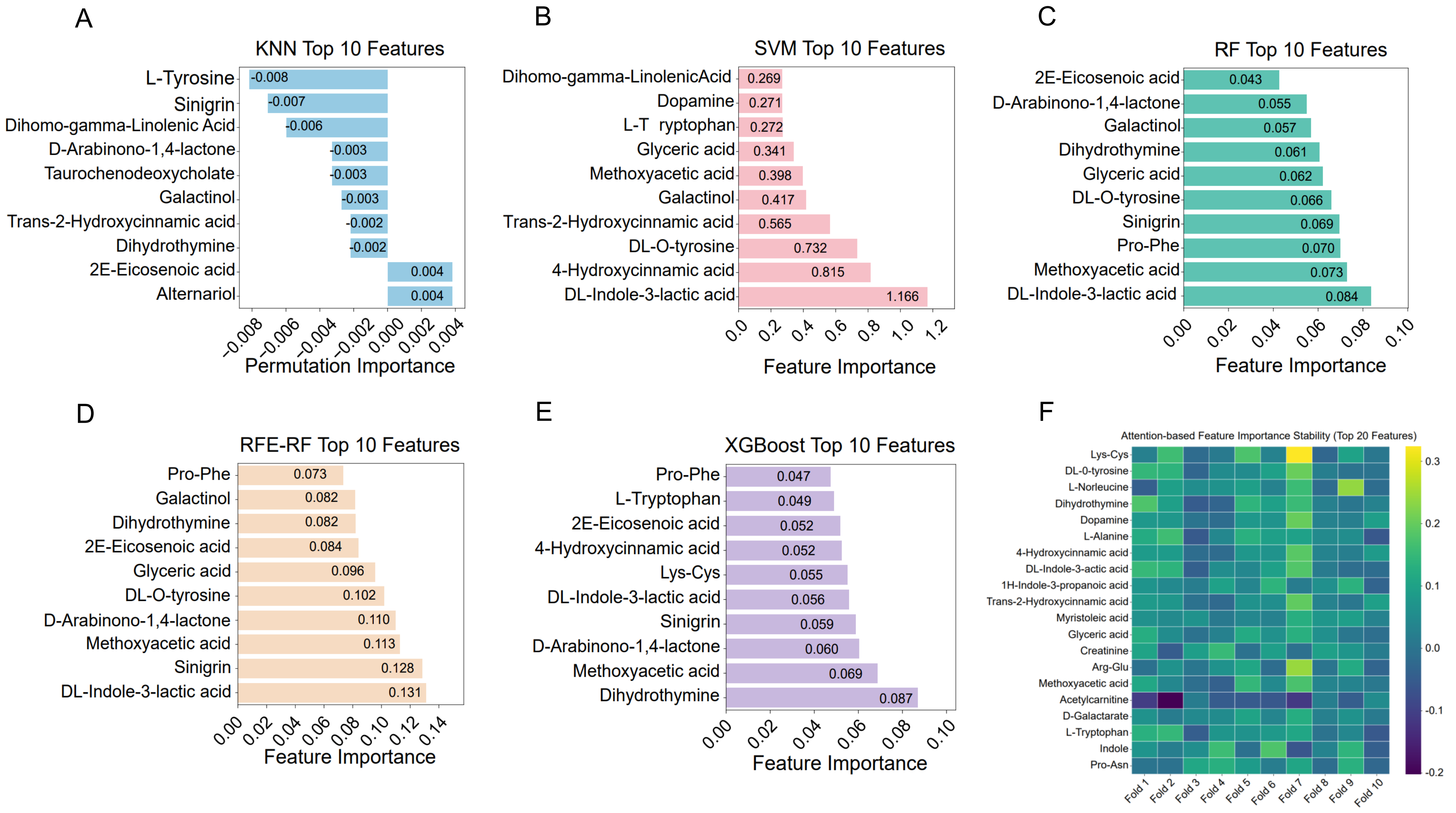

Supplementary Figure 3 Machine learning models look for important features. (A-E) The 25 differential metabolites shared by metabolomics and Transformer architecture were analyzed for KNN, RF, RFE-RF, SVM, and Top 10 features of XGBoost, respectively. (F) Attention heatmap of the top 20 metabolites obtained using the transformer architecture.

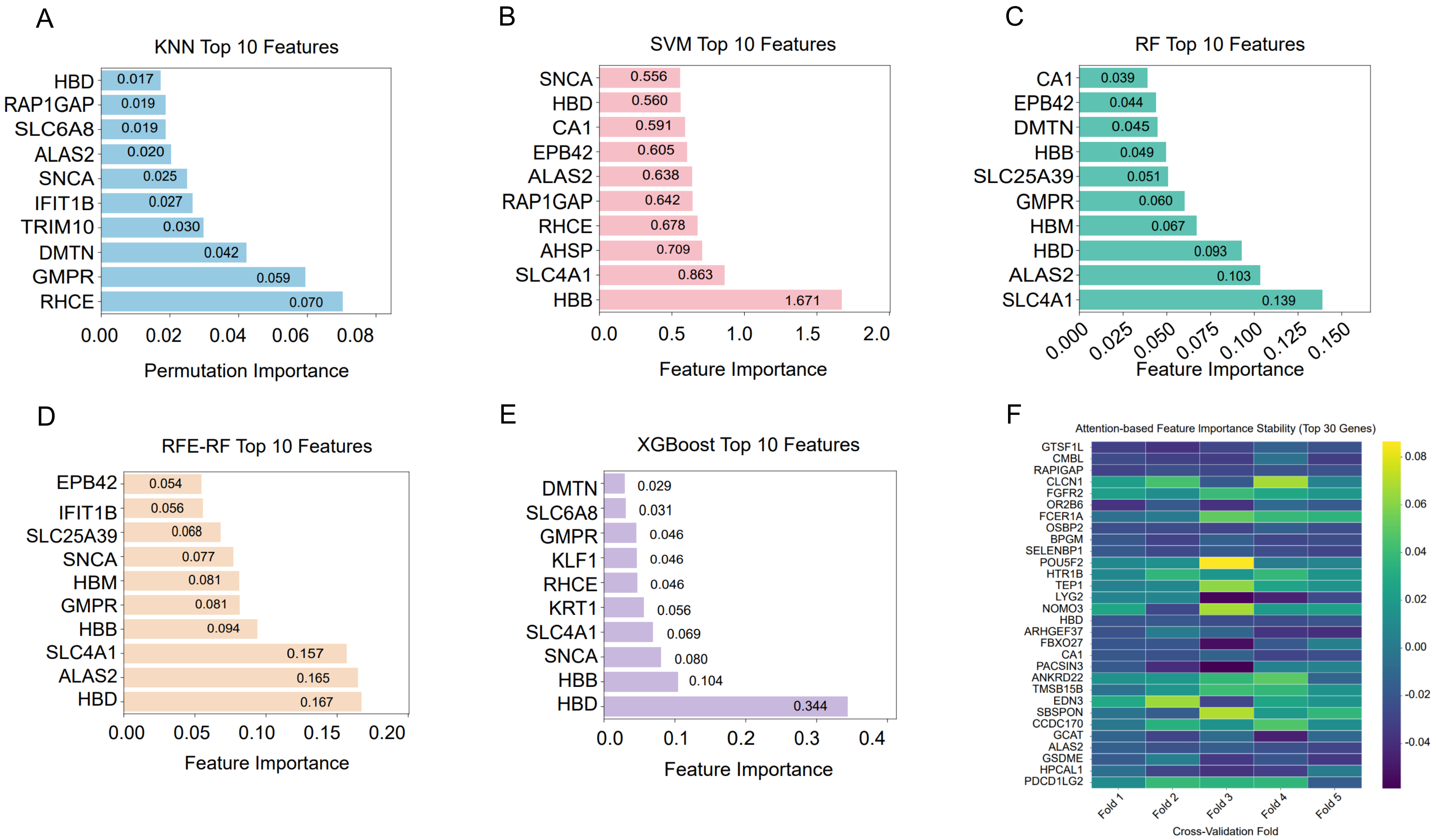

Supplementary Figure 4 Machine learning models look for important features. (A-E) The 25 differential metabolites shared by metabolomics and Transformer architecture were analyzed for KNN, RF, RFE-RF, SVM, and Top 10 features of XGBoost respectively. (F) Attention heatmap of the top 30 genes obtained using the transformer architecture.

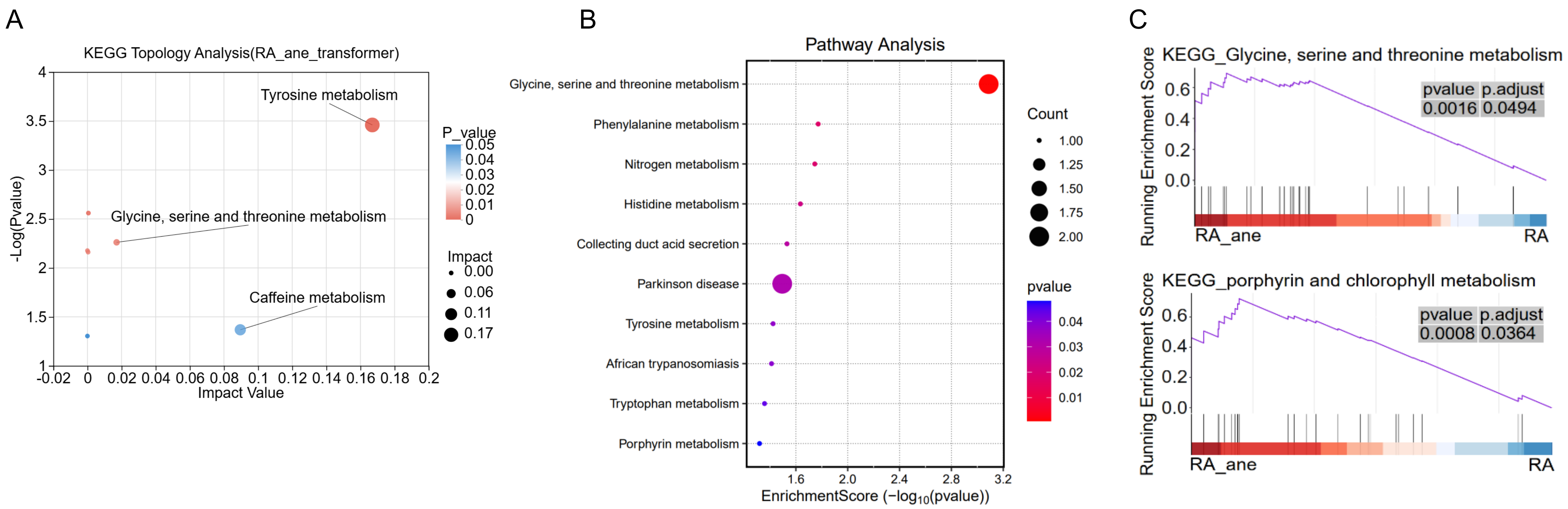

Supplementary Figure 5: Metabolomics and transcriptomics functional enrichment analysis. (A) KEGG topology analysis based on 25 differential metabolites shared by metabolomics and transformer structures. (B) KEGG enrichment analysis based on 55 differential genes shared by transcriptomics and transformer structures. (C) GSEA analysis of all detected genes by transcriptomics.

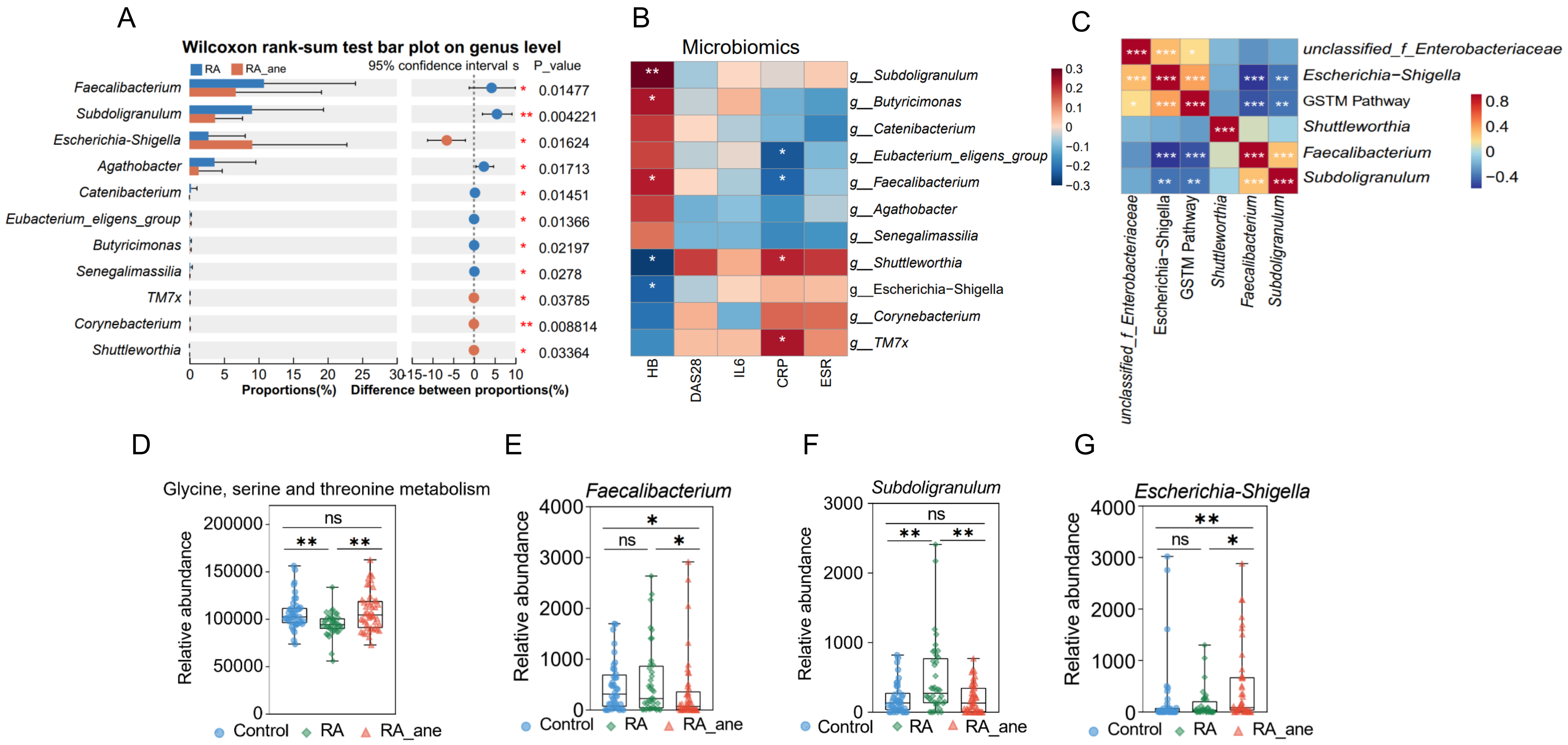

Supplementary Figure 6: Differential and functional analysis of dysbiotic intestinal flora of RA_ane. (A) There are 11 differential genera of RA_ane vs RA. Red, RA_ane group, blue, RA group; (B) Heat map of correlation between 11 different bacteria and HB and inflammation levels using *spearman* correlation analysis. Positive correlation coefficients are shown in red and negative correlation coefficients are shown in blue; (C) Correlation heatmap of the five different genera associated with HB with GSTM pathways; (D) Histogram of the abundance of GSTM pathways among the three groups; (E-G) Boxplot of the abundance of *Faecalibacterium, Subdoligranulum*, and *Escherichia-Shigella* between the three groups, using a two-by-two comparative non-parametric t-test for two-by-two comparisons. **P* <0.05, ***P* <0.01, ****P* < 0.001.

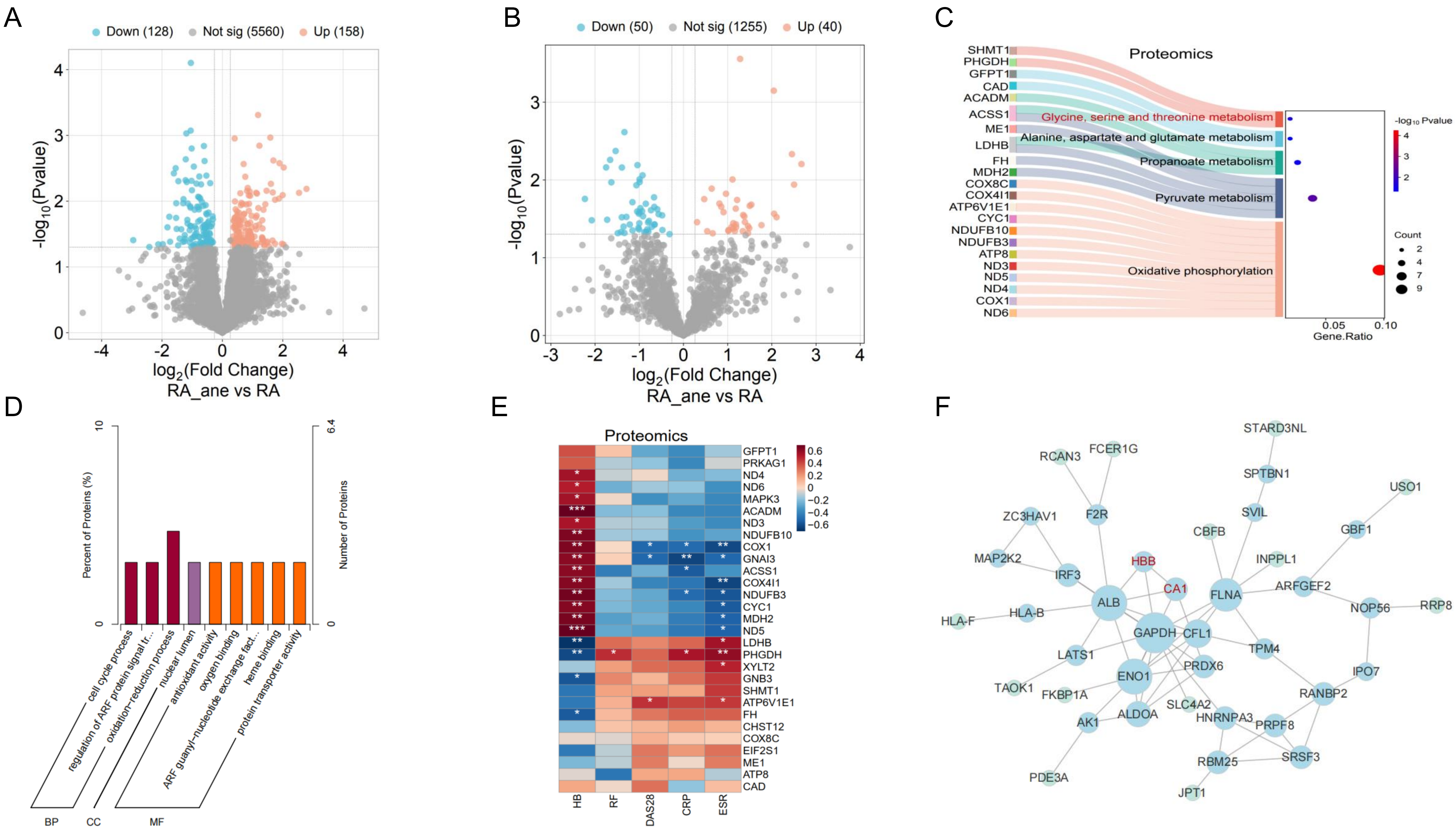

Supplementary Figure 7: Differential enrichment analysis of proteomics and phosphorylation proteomics of RA_ane. (A-B) Differential volcano plots of proteomics and phosphorylated proteomics between RA and RA_ane groups, respectively. (C) KEGG-enriched Sankey diagram of proteomics. (D) Phosphorylated proteomics GO enrichment analysis. (E) Heatmap of spearman correlation of genetically encoded proteins on the KEGG pathway of proteomics with HB, an indicator of inflammation. (F) PPI analysis of all genes encoding differentially phosphorylated proteins, the red-flagged CAI and HBB genes are also significantly different in transcriptomics

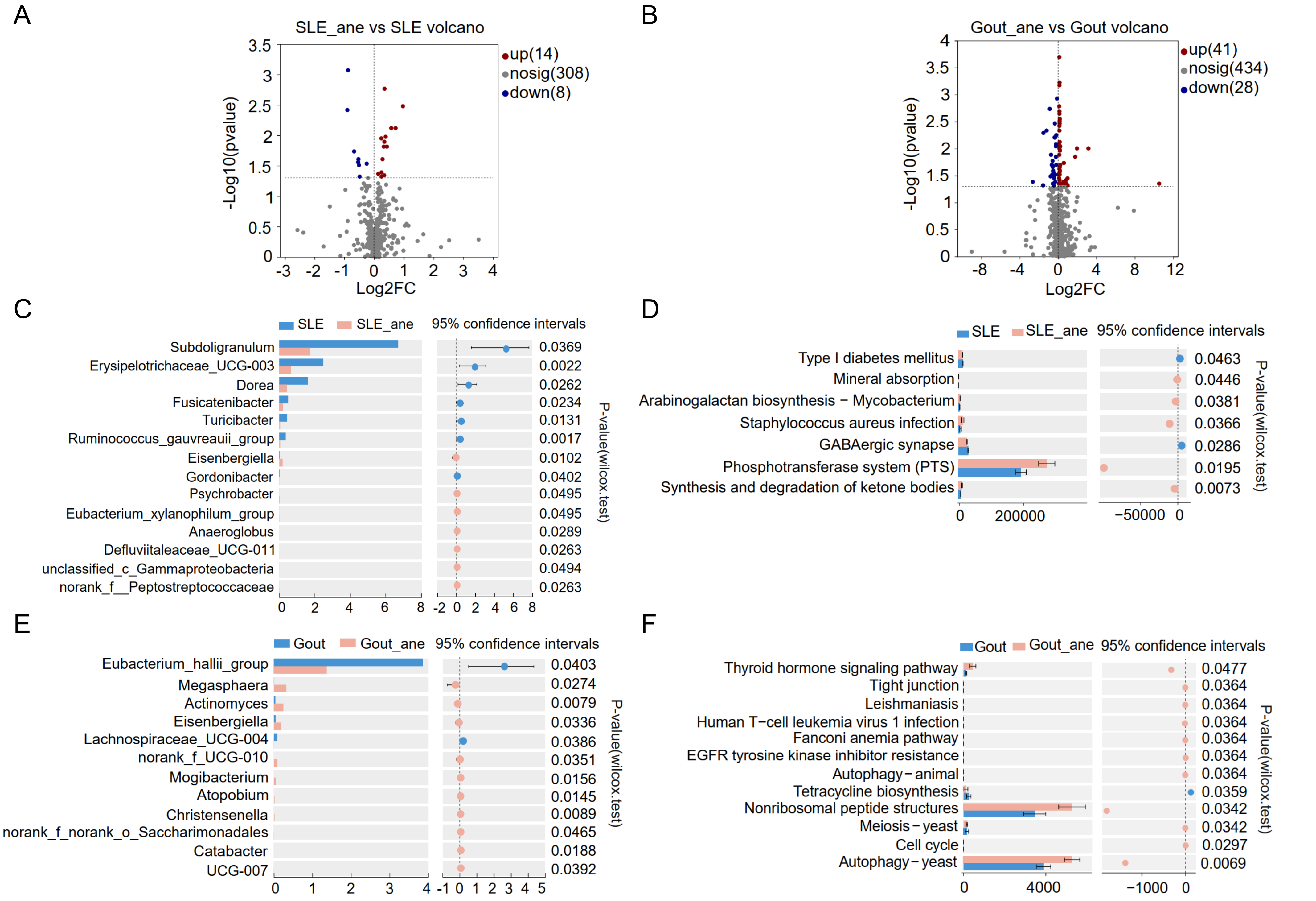

Supplementary Figure 8: Changes and functional prediction of serum metabolites and gut microbes in SLE_ane and gout_ane. (A) Volcano plot of differential metabolites between SLE and SLE_ane. (B) Volcano plot of differential metabolites between gout and gout_ane. (C) Differential bacterial genus histogram between SLE and SLE_ane obtained by wilcox' test method. (D) PICRUSt2 analysis of SLE and SLE_ane bacteria and associated enzyme pathway predictions. (E) Wilcox' test method to obtain differential genus histograms between gout and gout_ane. (F) PICRUSt2 analysis of bacterial and associated enzyme pathway predictions between gout and gout_ane.

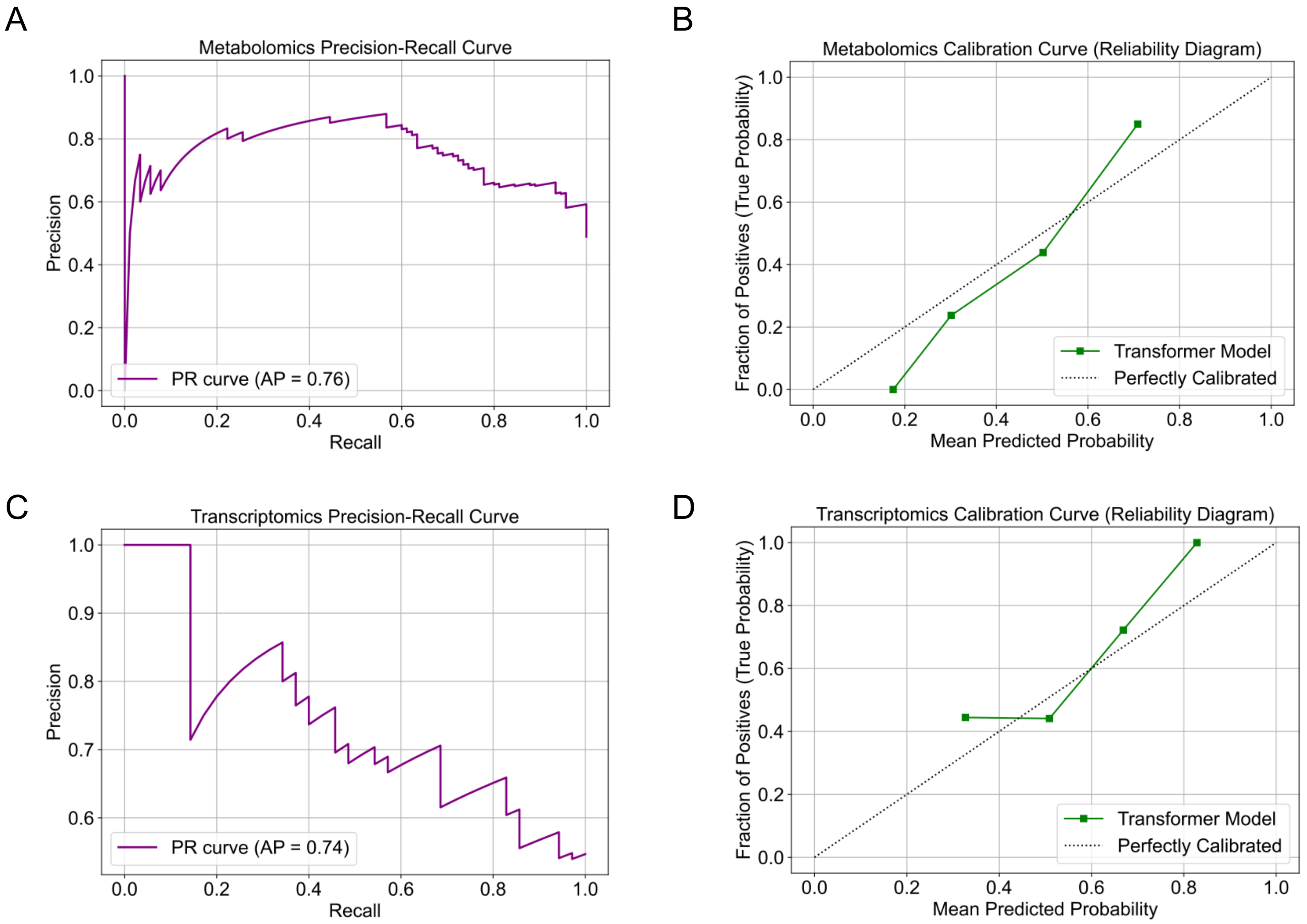

Supplementary Figure 9 Extended performance evaluation of the Transformer-based diagnostic models. (A) Precision-recall (PR) curve for the metabolomic model, with an average precision (AP) of 0.76. (B) Calibration curve (reliability diagram) for the metabolomic model, using 5 quantile bins (n_bins=5) given the moderate sample size. (C) PR curve for the transcriptomic model, achieving an AP of 0.74. (D) Calibration curve for the transcriptomic model, also with 5 bins. In the calibration plots, the dashed diagonal line indicates perfect calibration, and the solid curves represent the actual model performance across the binned predicted probabilities.

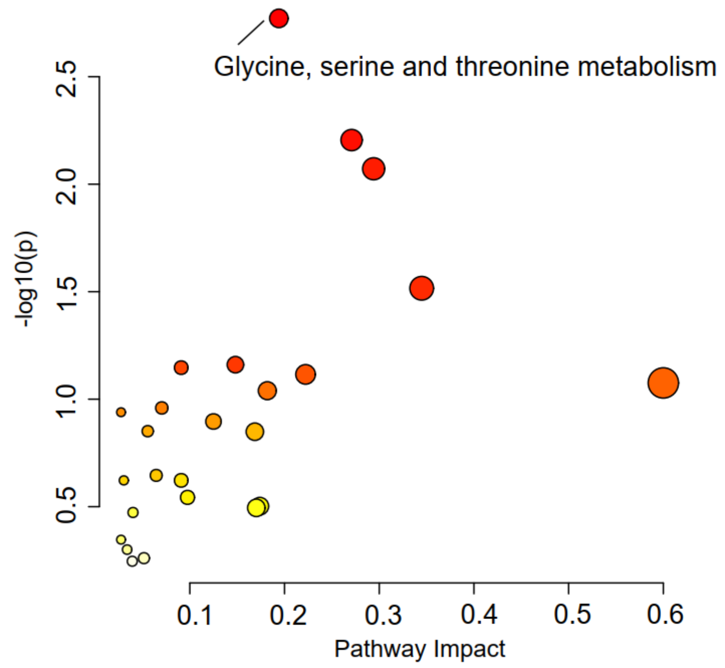

Supplementary Figure 10 Combined metabolomics and transcriptomics functional enrichment analysis of RA_ane.

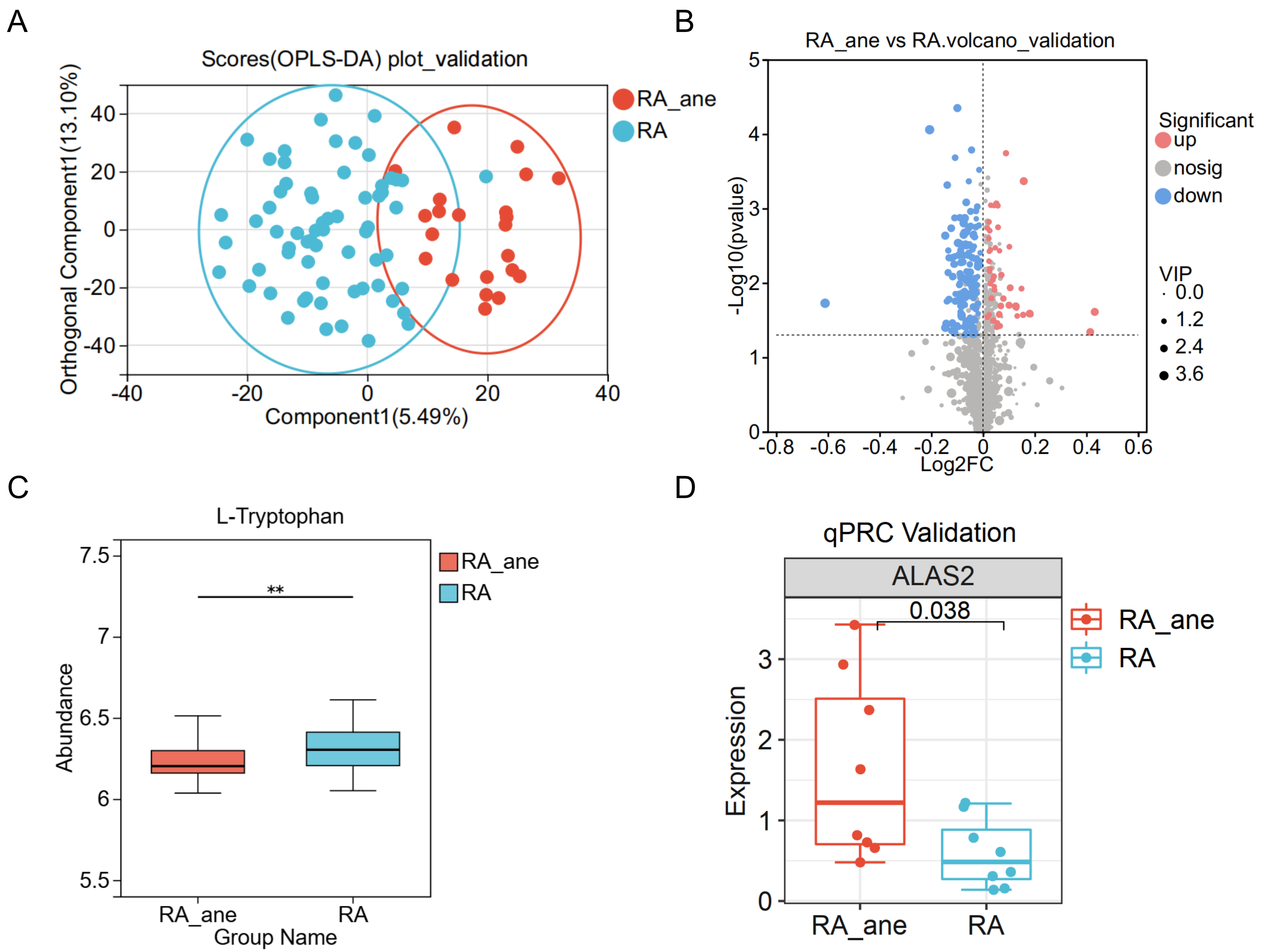

Supplementary Figure 11 Validation of the GSTM pathway. (A) OPLS-DA score plot of the metabolomics profile from the validation cohort, comparing RA_ane vs. RA groups. (B) Volcano plot of differentially abundant metabolites. (C) Box plot comparing the relative abundance of L-Tryptophan between groups. (D) Box plot of ALAS2 gene expression levels validated by qPCR.

Supplementary Table 1: Baseline characteristics of RA and Non-RA cohorts.

	**RA (n=4114)**	**Non-RA (n=100934)**
Mean age, years (S.D.)	55.8 ± 12.6	41.8 ± 16.3
Female(n,%)	3205 (77.9%)	46752 (46.3%)
HB(g/L, mean, S.D.)	123.1 ± 17.7	144.0 ± 16.7

Supplementary Table 1: Baseline characteristics of populations in the RA and Non-RA cohorts. Gender was expressed as a percentage, while age and HB were presented as mean ± standard deviation (SD).RA, rheumatoid arthritis cohort; Non-RA, non-rheumatoid arthritis cohort; HB, hemoglobin.

Supplementary Table 2: Clinical characteristics of RA and control populations.

Component	Control (n=113)	RA (n=127)	RA_ane (n=130)	P_value
Age	52.45±9.47	57.32±12.77	58.06±12.84	0.613
Female(%)	45.13%	76.38%	79.23%	0.582(χ²)
BMI	24.07±3.49	22.92±2.86	22.75±3.35	0.059
Smoking(%)		23.40%	17.78%	0.346(χ²)
Drinking(%)		20.21%	10.00%	0.054(χ²)
HB(g/L)	135.89±11.47	127.61±10.79	97.70±12.72	<0.001
RF(U/mL)		289.17±332.62	278.00±271.47	0.818
CRP(mg/L)		30.25±34.31	55.00±45.57	<0.001
ESR(mm/1h)		49.90±24.92	78.08±27.82	<0.001
IL6(pg/mL)		54.21±58.69	85.74±94.37	<0.001
DAS28		5.80±1.30	5.70±1.46	0.016
Comorbidity				0.590(Fisher's)
Gastrointestinal		15.75%	10.00%	
Renal		13.39%	13.08%	
Infection-related		2.36%	4.62%	
Multisystem		9.45%	8.46%	
Others		59.06%	63.85%	
Therapeutic				1.000(χ²)
csDMARDs		90.55%	90.77%	
Biologics & tsDMARDs		9.45%	9.23%	
Disease Duration				0.791(χ²)
≤ 2 years		37.01%	34.62%	
2 – 10 years		29.13%	33.08%	
> 10 years		33.86%	32.31%	
Omics				
Metabolomics	n=49	n=94	n=90	
Microbiomics	n=40	n=40	n=46	
Transcriptomics	n=24	n=29	n=35	
Proteomics		n=9	n=10	
Phosphorylated proteomics		n=4	n=6	

Supplementary Table 2: Clinical characteristics of RA and control population. Clinical data were expressed as mean ± SD and statistical significance was assessed using the unpaired wilcox t-test, categorical variables were analyzed using the chi-square test. Multi-omics populations are distributed at the end of the above table. *Comorbidities were categorized into mutually exclusive groups: gastrointestinal (chronic gastritis, peptic ulcer, inflammatory bowel disease, autoimmune gastritis); renal (chronic nephritis, diabetes, hypertension); infection-related (chronic infections including tuberculosis, hepatitis B/C, recurrent infections); multisystem (≥2 of the above categories); and others (none of the above). RA, rheumatoid arthritis; RA_ane, rheumatoid arthritis complicated by anemia; M/F, male/female; BMI, body mass index; HB, hemoglobin; RF, rheumatoid factors; CRP, C-reactive protein; ESR, erythrocyte sedimentation rate; IL6, interleukin-6; DAS28, disease activity score in 28 joints; csDMARDs, conventional synthetic disease-modifying antirheumatic drugs; tsDMARDs, targeted synthetic disease-modifying antirheumatic drugs.

Supplementary Table 3: Clinical characteristics of SLE and gout populations.

Component	Gout (n=19)	Gout_ane (n=18)	P_value	SLE (n=51)	SLE_ane (n=37)	P_value
Age	69.32±7.23	72.94±7.64	0.147	45.29±12.57	43.19±12.92	0.354
Female/Male	10.53%	5.56%	0.580(χ^2)	92.16%	91.89%	0.964(χ^2)
HB(g/L)	133.89±10.07	97.44±16.47	<0.001	128.16±11.89	95.41±12.09	<0.001
CRP(mg/L)	29.55±38.24	50.00±40.59	0.083	4.17±5.12	11.40±18.98	0.036
ESR(mm/1h)	29.53±18.45	68.71±34.20	<0.001	32.30±24.06	45.61±33.67	0.054
UA(umol/L)	475.58±150.06	486.50±131.48	0.504			
Metabolomics	n=18	n=18		n=33	n=15	
Microbiomics	n=17	n=17		n=27	n=29	

Supplementary Table 3: Clinical characteristics of RA and control population. Clinical data were expressed as mean ± SD and statistical significance was assessed using the unpaired wilcox t-test. Multi-omics populations are distributed at the end of the above table. RA, rheumatoid arthritis; RA_ane, rheumatoid arthritis complicated by anemia; M/F, male/female; BMI, body mass index; HB, hemoglobin; RF, rheumatoid factors; CRP, C-reactive protein; ESR, erythrocyte sedimentation rate; IL6, interleukin-6; DAS28, disease activity score in 28 joints.

Supplementary Table 4: Model Performance Comparison.

	Metabolomics					Transcriptomics				
	AUC	Accuracy	F1 Score	Precision	Recall	AUC	Accuracy	F1 Score	Precision	Recall
KNN	0.677	0.636	0.594	0.653	0.629	0.679	0.641	0.657	0.688	0.629
SVM	0.700	0.641	0.598	0.662	0.800	0.627	0.516	0.644	0.538	0.800
RF	0.702	0.647	0.611	0.662	0.771	0.752	0.750	0.771	0.771	0.771
RFE-RF	0.693	0.630	0.618	0.625	0.800	0.719	0.734	0.767	0.737	0.800
XGBoost	0.686	0.630	0.609	0.631	0.686	0.691	0.656	0.686	0.686	0.686

Supplementary Table 4: This table presents the performance metrics (mean values from five-fold cross-validation) of five machine learning models on the subset of their respective top 10 important features. All models demonstrated solid performance on both omics data types, supporting the subsequent comparative analysis of the feature sets. Metrics include the Area Under the Curve (AUC), Accuracy, F1 Score, Precision, and Recall. KNN, K-Nearest Neighbors; SVM, Support Vector Machine; RF, Random Forest; RFE-RF, Random Forest with Recursive Feature Elimination; XGBoost, eXtreme Gradient Boosting; AUC, Area Under the Curve.

Supplementary Table 5: Multivariate analysis of factors influencing hemoglobin levels in RA patients.

Variable	Linear Regression Coefficient	Ridge Regression Coefficient	Random Forest Importance	Effect Direction	Significance
ESR	-0.383	-11.339	0.640	Negative	***
DAS28	0.489	0.661	0.120	Positive	**
CRP	-0.003	-0.156	0.102	Negligible	**
Age	-0.054	-0.684	0.058	Negligible	*
Gender	-10.145	-4.208	0.057	Negative	*
Disease duration	-0.388	-0.280	0.020	Negative	---
Treatment	2.796	0.817	0.005	Positive	---

Supplementary Table 5: Results from multivariate analyses examining factors associated with hemoglobin levels in RA patients (N=257). Three analytical approaches were employed: linear regression (unstandardized coefficients), ridge regression (standardized coefficients), and random forest (feature importance scores). All models were adjusted for all variables listed. Significance levels based on random forest importance: *** $p<0.001$, ** $p<0.01$, * $p<0.05$. The overall model explained substantial variance in hemoglobin levels ($R^2=1.000$).
