# Supplementary material for: Transformer-Based Multiomics Study Identifies Important Role of Glycine, Serine, and Threonine Metabolism Pathway in Rheumatoid Arthritis Complicated by Anemia
Source: Comput Struct Biotechnol J. 2026 May 7;35(1):0075. doi: 10.34133/csbj.0075 (PMC13150071; doi:10.34133/csbj.0075)
Supplement: Supplementary 1 — Supplementary Materials 1 Supplementary Materials 2 Figs. S1 to S11 Tables S1 to S5 [file csbj.0075.f1.zip › Supplementary Material 1.docx]

**Title: Transformer-based multi-omics study identifies important role of glycine, serine and threonine metabolism pathway in rheumatoid arthritis complicated by anemia.**

**Authors: Jiaxin Huang1*, Yuanli Wei2*, Dongmei Wang2*, Jianghua Chen1, Congcong Jian3, Xiaoting Zhu4, Shilin Li4, Jie Zhang4, Tingting Wang2, Caizhen Liu2, Lingli Wei2, Jing Gao2, Jing Zhu5, Qinghua Zou6 #, Jianhong Wu2 #, Fanxin Zeng1,4#.**

**All methodological supplementary materials**

**Non-targeted metabolomics approaches**

**1. Metabolite Extraction**
Human plasma samples were thawed, and 100 μL aliquots were mixed with 400 μL of extraction solution (acetonitrile:methanol = 1:1, v/v) containing 0.02 mg/mL of internal standard (L-2-chlorophenylalanine). The homogenate was incubated at -20°C for 30 min to facilitate comprehensive protein precipitation. Following centrifugation at 13,000 × g at 4°C for 15 min, the supernatant was carefully transferred to sample vials for LC-MS/MS analysis. To rigorously monitor the stability and repeatability of the entire analytical workflow, pooled quality control (QC) samples were prepared by combining equal-volume aliquots from all biological samples. One QC sample was intercalated into the analytical sequence every 5-15 experimental samples.

**2. UHPLC-MS/MS Analysis**

Chromatographic separation of plasma metabolites was performed on a Thermo UHPLC system coupled with an ACQUITY UPLC HSS T3 column (100 mm × 2.1 mm i.d., 1.8 µm; Waters, Milford, MA, USA). The binary mobile phase system was precisely formulated as follows: solvent A consisted of 95% water and 5% acetonitrile (containing 0.1% formic acid), while solvent B comprised 47.5% acetonitrile, 47.5% isopropanol, and 5% water (containing 0.1% formic acid) [1]. A 2 µL sample aliquot was injected with a constant flow rate of 0.40 mL/min, and the column temperature was strictly maintained at 40°C. The optimized elution gradient was programmed as follows: 0–3 min, a linear decrease of phase A from 95% to 80% and a corresponding increase of phase B from 5% to 20%; 3–9 min, phase A decreased to 5% while phase B increased to 95%; 9–13 min, the gradient was held at 5% A and 95% B; 13.0–13.1 min, phase A was rapidly reverted to 95% and phase B to 5%, followed by an equilibration phase at 95% A and 5% B from 13.1 to 16 min. [2]. Mass spectrometric data acquisition was executed using a Thermo Q Exactive Mass Spectrometer equipped with an electrospray ionization (ESI) source operating in both positive and negative ion modes.

**3. Data Preprocessing, Annotation, and Quality Control**
Following data acquisition, raw LC-MS instrument files were imported into Progenesis QI software (Waters Corporation, USA) for comprehensive processing, including baseline filtering, peak recognition, integration, retention time (RT) correction, and peak alignment. To computationally mitigate potential inter-batch variations, global RT alignment and merged database searching were executed within the software environment. During preprocessing, metabolic features with > 20% missing values across samples were excluded, and residual missing values were imputed using the minimum observed value. Total sum normalization was subsequently applied to correct for any minor intra-batch signal drifts. To guarantee quantitative reliability, features exhibiting a relative standard deviation (RSD) > 30% in the QC samples were discarded, and the resulting robust matrix was log10-transformed.

For rigorous metabolite annotation, the MS1 precursor mass error tolerance was strictly constrained to < 10 ppm, with an MS/MS fragment tolerance of < 0.02 Da. Identifications achieving Metabolomics Standards Initiative (MSI) levels 1 and 2 were established through a comprehensive dual-scoring system: empirical Fragmentation Scores evaluated against an in-house standard library, Metlin, and LipidBlast, supplemented by Theoretical Fragmentation Scores matched against the HMDB database.

**4. Multivariate Statistical Analysis and Feature Consensus**Multivariate analyses of the preprocessed metabolomics matrix were conducted using the *ropls* package (Version 1.6.2) in R. Principal component analysis (PCA) was initially employed to assess overall unsupervised distribution and detect outliers, followed by orthogonal partial least squares discriminant analysis (OPLS-DA) to maximize group separation. The robustness of the OPLS-DA model was evaluated via 7-fold cross-validation. Significantly altered metabolites were defined by meeting a composite criterion: an OPLS-DA variable importance in projection (VIP) score > 1.0 and a nominal P-value < 0.05 derived from Student's t-test (or the Wilcoxon rank-sum test for non-normally distributed data). Notably, applying strict univariate FDR correction (e.g., Benjamini-Hochberg) to untargeted metabolomics frequently incurs severe false-negative rates due to the high collinearity and non-independence of metabolic features within interconnected biological networks. Therefore, to comprehensively guard against false positives, this initial statistical screening was strictly coupled with an algorithmic consensus filter. Specifically, the identified differential metabolites were intersected with the core features extracted from our deeply regularized Hybrid PCA-Transformer machine learning architecture (validated via rigorous pooled cross-validation). This "Statistical + Algorithmic Consensus" dual-filter approach ensures the universal stability and algorithm-agnostic nature of the final biomarker panel. Finally, pathway enrichment analysis of these highly robust metabolites was executed via the *scipy.stats* package in Python, utilizing Fisher's exact test mapping against the KEGG database (<https://www.kegg.jp>) to identify significantly disrupted metabolic networks [3].

**Methods for intestinal bacteria analysis**

**1.Sample Collection**

The first fecal samples of all participants were collected and registered for laboratory processing, and the fecal samples were divided according to the standard procedure (at least 1ml for each EP tube), and the separated EP tubes were quickly frozen in liquid nitrogen tanks for 1 minute, and then transferred to the refrigerator at -80℃ for storage.

**2.DNA extraction and PCR amplification**

Total DNA extraction from fecal samples was performed using the E.Z.N.A.® soil DNA kit (Omega Bio-tek, Norcross, GA, U.S.) kit and according to its instructions. The quality of all DNA samples was checked by 1% agarose gel electrophoresis, and DNA concentration and purity were determined using a NanoDrop2000. Primers 338F (5'-ACTCCTACGGGGAGGCAGCAG-3') and 806R (5'-GGACTACHVGGGGTWTCTAAT-3') were used to analyze the V3-V4 variable region PCR of the bacterial 16S rRNA gene. V3-V4 variable region of bacterial 16S rRNA gene was amplified by PCR, and ABI GeneAmp® 9700 was selected as the PCR instrument.

The PCR reaction system was as follows: 4 μL of 5×TransStart FastPfu buffer, 2 μL of 2.5 mM dNTPs, 0.8 μL of upstream primer (5 uM), 0.8 μL of downstream primer (5 uM), 0.4 μL of TransStart FastPfu DNA polymerase, 10 ng of template DNA, and 20 μL of ddH2O. 3 replicates were performed for each sample. 3 replicates per sample.

**3.Illumina MiSeq sequencing**

PCR products were recovered using a 2% agarose gel, purified according to the instructions of the AxyPrep DNA Gel Extraction Kit (Axygen Biosciences, Union City, CA, USA), and quantified by Quantus™ Fluorometer (Promega, USA). The products were quantified by Quantus™ Fluorometer (Promega, USA). Then library construction was performed NEXTflexTM Rapid DNA-Seq Kit (Bioo Scientific, USA), (1) splice linkage; (2) removal of splice self-linking fragments using magnetic bead screening; (3) enrichment of library templates using PCR amplification; and (4) magnetic bead recycling of PCR products to obtain the final library. Sequencing was performed using Illumina's Miseq PE300 platform (Shanghai Meiji Biomedical Technology Co., Ltd.).

**4.Processing of sequencing data**

The raw sequenced sequences were first preprocessed for quality control using FASTP software (https://github.com/OpenGene/fastp,version 0.20.0), and then spliced using FLASH (http://www.cbcb.umd.edu/software/flash, version 1.2.7) software for splicing, the process was as follows:

① Filter the bases with quality value of 20 or less at the end of the reads, set a window of 50 bp and filter the reads with 50 bp or less after quality control, and remove the reads containing N bases;

② Splicing pairs of reads into one sequence according to the overlap relationship between PE reads;

③ Screening sequences that do not meet the maximum mismatch ratio of 0.2;

④ Distinguish the samples according to the barcode and primers at the beginning and end of the sequence, and adjust the sequence orientation, with no mismatches allowed for the barcode and the number of primer mismatches allowed to be 2.

Based on the default parameters, the optimized sequences after QC splicing were noise-reduced to obtain ASVs (Amplicon Sequence Variants) using the DADA2 plug-in in the Qiime2 process. The number of noise-reduced sequences for each sample was drawn flat and next compared with the Silva 16S rRNA database (version 138) with a threshold of 70%. Finally, it was performed through the diversity cloud analysis platform (Qiime2 process) (cloud.majorbio.com) of Shanghai Major Biomedical Technology Co. for subsequent data analysis, during analysis, we determined groupings and performed analyses using the minimum number of sequences per sample [3-5].

**DIA quantitative proteomic analysis method**

**1.Total Protein Extraction**

Peripheral venous blood samples were collected from the participants, using whole blood samples added to red blood cell lysate and then a series of standard laboratory procedures to obtain leukocyte samples, which were frozen at -80°C in a refrigerator. The samples were removed from the refrigerator, transferred to a 1.5 ml centrifuge tube, and lysed by adding an appropriate amount of DB proteolytic solution (8 M urea, 100 mM TEAB, pH=8.5). After centrifugation, the supernatant was alkylated with 1M DTT for 1 h at room temperature with sufficient iodoacetamide and protected from light [6, 7].

**2. Protein Quality Inspection**

Use Bradford protein quantification kit to prepare BSA standard protein solution according to the instructions. The BSA standard protein solution with different concentration gradients and the sample solution to be tested with different dilutions were put into 96-well plates, and the same concentration gradients were repeated 3 times. Quickly add 180 µL of G250 staining solution, let it stand at room temperature for 5 min, and measure the absorbance at 595 nm. After plotting the standard curve, the protein concentration of the samples to be tested was calculated. 20µg of each protein sample was subjected to 12% SDS-PAGE gel electrophoresis at 80 V, 20 min for the concentration gel and 120 V, 90 min for the separation gel, and then stained with Cauloblue R-250 and decolorized until the bands were clear.

**3.Trypsin treatmen**t

Each protein sample was taken and digested with a mixture of DB lysis buffer (8 M urea, 100 mM TEAB, pH=8.5), trypsin and 100 mM TEAB buffer for 4 h at 37°C, followed by the addition of trypsin and CaCl2 digestion overnight. Formic acid was added to adjust pH < 3. After centrifugation at room temperature, the supernatant was slowly passed through a C18 desalting column, after which it was washed three times consecutively with washing solution (0.1% formic acid, 3% acetonitrile), and then appropriate amount of eluent (0.1% formic acid, 70% acetonitrile) was added, and the filtrate of the samples was collected and lyophilized [8].

**4. DDA Spectral Library Construction and DIA LC-MS/MS Analysis**
Proteomic data acquisition was performed at Novogene Co., Ltd. (Beijing, China) utilizing a nanoElute nanoscale UHPLC system coupled to a timsTOF Pro 2 mass spectrometer (Bruker Daltonics). Peptides were separated on a high-resolution analytical column (25 cm × 75 μm, 1.6 μm particle size) prior to mass spectrometric evaluation. To construct the comprehensive Data-Dependent Acquisition (DDA) spectral library, the mass spectrometer was operated with a Captive Spray ionization source (spray voltage set to 1.5 kV). Following library generation, the individual biological samples were analyzed under Data-Independent Acquisition (DIA) mode using identical chromatographic conditions. For DIA scans, the full mass acquisition range was set from *m/z* 100 to 1700, with a ramp time of 100 ms and a Lock Duty Cycle of 100%. The DIA isolation scheme was configured with a scanning window size of 25 Da and 2 mobility windows, generating the raw data (.d) files for subsequent quantitative processing [9].

**5. Proteomic Data Processing, Quality Control, and Statistical Analysis**
The raw mass spectrometry data (.d files) were analyzed using Spectronaut software (Biognosys). Initially, the Pulsar module was employed to search the DDA raw files against the reference protein database to construct a highly confident target spectral library. For quality control at the identification level, a strict target-decoy search strategy was enforced: both the peptide-spectrum match (PSM)/peptide-level and the protein-level False Discovery Rates (FDR) were strictly thresholded at < 1.0% (0.01). Only proteins fulfilling this stringent 1% FDR criterion were retained.

Subsequently, the DIA raw data were mapped against this DDA library for targeted data extraction and protein quantification. Retention time alignment across all sample runs was automatically executed by Spectronaut utilizing an indexed Retention Time (iRT) standard system, driven by non-linear regression and a dynamic local calibration model. To preserve the authentic biological variance of low-abundance proteins in the leukocyte samples, no missing value imputation was performed. Relative protein quantification was achieved by extracting and integrating precursor ion peak areas.

For the identification of differentially expressed proteins (DEPs), quantitative comparisons between groups were conducted using Student's t-tests. To balance discovery sensitivity with analytical rigor in the inherently constrained proteomic dataset, DEPs were defined based on a nominal *P*-value < 0.05 combined with a fold-change threshold of ≥ 1.2 (for up-regulation) or ≤ 0.83 (for down-regulation). The statistically significant DEPs were subsequently subjected to systematic functional characterization, including mapping to the COG, GO, and KEGG databases for pathway enrichment and protein-protein interaction network topology analysis [10-12].

**Phosphorylated proteomic analysis method**

**1.Total Protein Extraction**

Peripheral venous blood samples were collected from the participants, using whole blood samples added to red blood cell lysate and then a series of standard laboratory procedures to obtain leukocyte samples, which were frozen at -80°C in a refrigerator. The samples were removed from the refrigerator, transferred to a 1.5 ml centrifuge tube, and lysed by adding an appropriate amount of DB proteolytic solution (8 M urea, 100 mM TEAB, pH=8.5). After centrifugation, the supernatant was alkylated with 1M DTT for 1 h at room temperature with sufficient iodoacetamide and protected from light [6, 7].

**2. Protein Quality Inspection**

Use Bradford protein quantification kit to prepare BSA standard protein solution according to the instructions. The BSA standard protein solution with different concentration gradients and the sample solution to be tested with different dilutions were put into 96-well plates, and the same concentration gradients were repeated 3 times. Quickly add 180 µL of G250 staining solution, let it stand at room temperature for 5 min, and measure the absorbance at 595 nm. After plotting the standard curve, the protein concentration of the samples to be tested was calculated. 20µg of each protein sample was subjected to 12% SDS-PAGE gel electrophoresis at 80 V, 20 min for the concentration gel and 120 V, 90 min for the separation gel, and then stained with Cauloblue R-250 and decolorized until the bands were clear [13].

**3.Proteolysis**

Take 5 mg of each protein sample, add protein lysis solution to make up the volume to 1 mL, add 50 ug of trypsin and 10 mL of 50 mM TEAB buffer, mix well, then add 50 ug of trypsin and CaCl2 enzyme digestion overnight. Add 50 ug of trypsin and 10 mL of 50 mM TEAB buffer, mix well and incubate at 37°C for 4h, and then add 50 ug of trypsin and CaCl2 for overnight digestion. Add formic acid to adjust the pH < 3. After centrifugation at room temperature, the supernatant was slowly passed through a C18 desalting column, and then washed with washing solution (0.1% formic acid, 3% acetonitrile) for three times, and then added appropriate amount of eluent (0.1% formic acid, 70% acetonitrile), and then collected the filtrate of the samples and lyophilized.

**4.Enrichment of phosphorylated modified polypeptides**

Add binding buffer to dissolve the lyophilized powder and centrifuge at 12000 g for 5 min at 4°C. Add the supernatant to the IMAC-Fe column pre-treated with binding buffer and incubate for 30 min at room temperature. The supernatant was added to an IMAC-Fe column pre-treated with binding buffer and incubated for 30 min at room temperature. After centrifugation at 2000 g for 30 s, the column was washed once with washing solution and once with water, and then centrifuged at 2000 g for 30 s. The tubes were disposed of and replaced with new tubes with the eluent solution, and the peptide eluent was collected and lyophilized.

**5. DDA spectrum library construction**

DDA spectrum library construction and DIA mode identification using UHPLC-MS/MS were

performed in Novogene Co., Ltd. (Beijing, China). Fractions were separated using a nanoElute nanoscale UHPLC system with an analytical column (25 cm × 75 μm, 1.6 μm), tims TOF pro2 mass spectrometer, a Captive Spray ion source, and an ion spray voltage set to 1.5 kV to generate mass spectrometry detected raw data (. d) for construction of the DDA spectral library.

**6. LC-MS/MS Analysis-DIA mode**

A nanoElute nanoscale UHPLC system was used with an analytical column (25 cm×75 μm, 1.6 μm), a tims TOF pro2 mass spectrometer, a Captive Spray ionization source, a set ionization spray voltage of 1.5 kV, a full scanning range of the mass spectrometer of m/z 100-1700, a Ramp time of 100 ms, a Lock Duty Cycle to 100%, a scanning window size of 25 Da, and a trickle window of 2. Raw data (. d) were generated for the mass spectrometry. Duty Cycle to 100%; scanning window size 25Da, number of trickle windows 2. Raw data (. d) was generated for mass spectrometry [9].

**7. Data analysis**

On the one hand, the Raw files of the downlinked spectrograms acquired by DDA scanning were searched and solved by Spectronaut to convert the spectrogram information into protein information, and at the same time, quality control of the DDA data was carried out, and the quality-controlled data were imported into Spectronaut to construct a real spectrogram library, and on the other hand, the Raw files of the downlinked spectrograms acquired by DIA scanning and DDA library were compared with each other in Spectronaut for identification of the modified proteins. On the other hand, Spectronaut can compare the Raw files of the downstream spectra acquired by DIA scanning with the DDA library to identify the modified proteins.

Significantly differentially expressed proteins were screened according to the criterion of a 1.2-fold or greater fold change in expression (up-regulation of more than 1.2-fold or down-regulation of less than 0.50-fold) with a p-value< 0.05. The identified proteins were annotated with functional databases, including COG database, GO database and KEGG database; after that, we carried out the analysis of phosphorylation modification sites, quantitative analysis of proteins, differential analysis of phosphorylation modification proteins, as well as screening of differential proteins and clustering analysis of expression patterns; and finally, we carried out GO, KEGG functional enrichment analysis and interoperability network analysis for the differential proteins that were screened, and so on [10-12].

**RNA sequencing analysis method**

**1.Sample Collection Processing**

Treated human peripheral venous blood samples were collected to obtain at least one tube of leukocyte samples containing trizol reagent, which were frozen at -80°C in a freezer and sent to the company for sequencing. Total RNA extraction and detection were performed using Agilent 2100 bioanalyzerRNA.

**2. Library construction and quality control**

The starting RNA for library construction was total RNA, and mRNA with polyA tail was enriched by Oligo(dT) magnetic beads, followed by random interruption of the obtained mRNA with divalent cations in Fragmentation Buffer. The first strand of cDNA was synthesized in M-MuLV reverse transcriptase system using the fragmented mRNA as template and random oligonucleotides as primers, followed by degradation of the RNA strand by RNaseH and synthesis of the second strand of cDNA from dNTPs in DNA polymerase I system. The purified double-stranded cDNA was end-repaired, A-tailed and ligated into sequencing junctions, and the cDNA of about 370-420 bp was screened with AMPure XP beads, amplified by PCR, and the PCR products were purified with AMPure XP beads again to obtain the final library. After the library was constructed, Qubit 2.0 Fluorometer was used for initial quantification, and the library was diluted to 1.5 ng/ul. The insert size of the library was measured using an Agilent 2100 bioanalyzer, and after the insert size was as expected, qRT-PCR was used to accurately quantify the effective concentration of the library (the effective concentration of the library was higher than 1 ng). After the insert size met the expectation, qRT-PCR was used to accurately quantify the effective concentration of the library (the effective concentration of the library was higher than 2nM) to ensure the quality of the library [14].

**3. Computer sequencing**

After passing the library inspection, different libraries were pooled according to the effective concentration and the target downstream data volume and sequenced with Illumina NovaSeq 6000, and 150 bp paired-end reads were generated. Four types of fluorescently labeled dNTP, DNA polymerase, and junction primers are added to the sequencing flow cell for amplification. When extending the complementary strand of each sequencing cluster, each fluorescently labeled dNTP added releases corresponding fluorescence, which is then captured by the sequencer, and converted into sequencing peaks by the computer software, thus obtaining the sequence information of the fragment to be tested [15].

**4. Data quality control**

Sequenced fragments are converted into sequence data (reads) by CASAVA Base Recognition of the image data measured by a high-throughput sequencer. The raw data obtained from sequencing contains a small number of reads with sequencing adapters or low-quality sequencing. reads with adapters are removed, reads with N (N means that the base information cannot be determined) are removed, and low-quality reads (reads with Qphred <=20 that account for more than 50% of the total length of the read) are removed. High-quality analyses were performed based on clean data, with reference genome and gene model annotation files downloaded directly from the genome website. The reference genome was indexed and paired-end clean reads were compared to the reference genome using HISAT2 (v2.0.5). featureCounts (1.5.0-p3) was used to calculate the number of reads mapped to each gene.

**5. Data analysis**

We used the R package DESeq2(version 1.32.0) to perform the difference analysis in order to obtain the differential genes between the different comparison groups and the control group. Specifically,we obtained the expression profile dataset,removed the genes whose expression value was 0 with a proportion greater than 50%,constructed the input matrix using the DESeqDataSetFromMatrix function,and further normalized the data using the The input matrix was constructed using the DESeqDataSetFromMatrix function, the data were further normalized using the DESeq 2 function, and finally the difference analysis was performed using the results function to finally obtain the significance of the differences of each gene. Differentially expressed genes (DEGs): FDR<0.01, |Fold Change|>2, differentially expressed proteins and phosphorylated proteins: P<0.05, |Fold Change|>1.2. The statistical enrichment of differentially expressed genes in KEGG pathways was analyzed using clusterProfiler (3.8.1) software.

Tips: Metabolomics and Microbiome: Sequenced together at Majorbio using Vanquish H/Q-Exactive HF and Illumina NovaSeq6000 PE250 platforms, respectively. Transcriptomics, Proteomics and Phosphoproteomics: Processed together at Novogene using Illumina NovaSeq6000 (transcriptomics) and BRUKER TIMS PRO2 (proteomics) platforms. Since each omics dataset was generated in a single sequencing batch, inter-batch correction was not required.

**Quantitative PCR (qPCR) Validation**

The expression level of the key gene ALAS2 was experimentally validated using quantitative reverse transcription PCR (qRT-PCR). Total RNA was extracted from peripheral blood mononuclear cells (PBMCs) and whole blood samples obtained from a cohort of 10 RA_ane patients and 10 matched non-anemic RA controls, using a Blood RNA Extraction Kit (biosharp, BL1532A) according to the manufacturer's protocol. Genomic DNA was removed using the PrimeScript™ RT reagent Kit with gDNA Eraser (Takara Bio, RR047A). Reverse transcription was performed to synthesize cDNA under the following thermal conditions: 37°C for 15 min, followed by 85°C for 5 sec.

qPCR was carried out on a QuantStudio™ 3 Real-Time PCR System (ThermoFisher) using TB Green® Premix Ex Taq™ II Fast qPCR mix (Takara Bio, CN830A). The reaction mixture (25 µL) contained 12.5 µL of TB Green Premix, 1 µL each of forward and reverse primers (10 µM), 2 µL of cDNA template, and 8.5 µL of ddH₂O. The thermal cycling protocol consisted of an initial denaturation at 95°C for 1 min, followed by 45 cycles of 95°C for 10 s, 55°C for 30 s, and 72°C for 10 s. Gene-specific primers for ALAS2 (forward: 5′-CCTAGCTCCCTGGTCTCAGT-3′; reverse: 5′-GGTGTGTGACCTTCCCAGAG-3′) and the reference gene GAPDH (forward: 5′-TGACTTCAACAGCGACACCCA-3′; reverse: 5′-CACCCTGTTGCTGTAGCCAAA-3′) were used. The relative expression levels of ALAS2 were calculated using the 2^–ΔΔCt method.

**Machine Learning Methodology**

In this study, we employed a comprehensive machine learning framework to analyze and classify the transcriptomic data, with a particular focus on the Transformer architecture and other traditional machine learning models to identify differentially expressed genes and their predictive power in distinguishing between comparison groups and controls.

**Detailed Methodology for the Transformer-Based Diagnostic Models**

To ensure complete transparency and reproducibility, the following sections detail the architecture, data processing, hyperparameter configurations, and evaluation metrics for the Transformer-based machine learning models applied to both transcriptomic and metabolomic datasets.

1. **Data preprocessing.**

To appropriately format the input data for the Transformer architecture, the raw omics matrices were transposed so that each sample was represented as a sequence of molecular features. To mitigate the curse of dimensionality and ensure compatibility with the multi‑head attention mechanism, the data underwent standard z‑score normalization followed by principal component analysis (PCA). Crucially, to prevent information leakage, both scaling and PCA transformations were fitted exclusively on the training folds and then applied to the validation folds. The feature space was reduced to 10 principal components for the metabolomic dataset and 30 principal components for the transcriptomic dataset.

**2. Transformer encoder architecture.**

The core of our analytical framework is a three‑layer Transformer encoder designed to capture complex, non‑linear interactions among molecular features. To accommodate the distinct complexities and dimensionalities of the two omics layers, the multi‑head self‑attention mechanisms were tailored accordingly: the encoder was configured with 2 attention heads for the metabolomic data and 6 attention heads for the transcriptomic data. The sequence output from the encoder was then aggregated and passed through a final fully connected linear layer to generate a continuous logit representing the likelihood of the sample belonging to the anemic phenotype. To structurally prevent overfitting given the limited cohort sizes, substantial regularization was enforced in both models using a dropout rate of 0.5.

**3. Training procedure.**

The models were optimized using binary cross‑entropy with logits loss. We employed the Adam optimizer together with an L2 weight decay penalty of 0.1 to strictly constrain model complexity. The learning rate was set to 0.0005 for the metabolomic model and 0.0001 for the transcriptomic model. To halt training dynamically and avoid memorization of the training subsets, an early stopping mechanism was implemented. Specifically, training was terminated if the validation loss failed to improve over a predefined patience window: 8 epochs (maximum 80 training epochs) for the metabolomic model and 10 epochs (maximum 100 training epochs) for the transcriptomic model.

**4. Performance evaluation.**

To ensure robustness and generalizability, model performance was assessed using a rigorous stratified cross‑validation strategy: 10 folds for the metabolomic analysis and 5 folds for the transcriptomic analysis. Beyond the conventional area under the receiver operating characteristic curve (AUC‑ROC), our expanded evaluation framework comprehensively reported accuracy, precision, recall, and F1‑score. In addition, precision‑recall (PR) curves and calibration curves were generated to rigorously evaluate positive predictive precision and probability calibration for both omics modalities.

**5. Model interpretability.**

To interpret the biological relevance of the model decisions, we extracted the attention weights learned by the Transformer. These weights quantitatively indicate the relative contribution of each principal component, allowing us to project the predictive signals back to the original molecular features and identify the core biological drivers that most significantly contributed to the classification task.

**Traditional Machine Learning Framework**

In addition to the Transformer-based architecture, we rigorously evaluated five traditional machine learning algorithms to benchmark predictive performance and identify key discriminative features. To strictly prevent data leakage and ensure robust generalization, all conventional models were evaluated using a 5-fold stratified cross-validation framework across both the metabolomic and transcriptomic datasets. Within each cross-validation fold, continuous variables were normalized using standard scaling (z-score) fitted exclusively on the training subset to avoid information spillover into the validation set. Given the identical algorithmic configurations applied to both omics modalities, the following hyperparameters and feature evaluation strategies were consistently utilized.

**1. K-Nearest Neighbors (KNN)**

The KNN model was implemented to evaluate the discriminative capacity of the omics profiles based on local data topology. To mitigate susceptibility to baseline noise inherent in high-dimensional omics data and prevent overfitting on restricted sample sizes, the neighborhood size was conservatively set to five neighbors. Because the KNN algorithm lacks an inherent feature weighting mechanism, feature importance was dynamically derived using a permutation importance algorithm configured with 10 repetitions. This method effectively quantifies the relative importance of each molecular feature by measuring the decrease in model performance when the specific feature values are randomly shuffled.

**2. Support Vector Machine (SVM)**

A linear kernel SVM was deployed to define an optimal hyperplane maximizing the classification margin between the control and anemic cohorts. To ensure robust generalization and prevent overfitting, the regularization parameter (C) was strictly constrained to 1.0, allowing for a broader margin that tolerates minor noise in the clinical omics data. The model was mathematically configured to output precise probability estimates for downstream comparative evaluation. Feature importance within the SVM framework was directly inferred from the absolute values of the linear coefficients assigned to each underlying molecular feature.

**3. Random Forest (RF)**

An ensemble learning approach using Random Forest was employed to capture complex non-linear interactions among molecular features. To structurally prevent algorithmic memorization, the complexity of individual decision trees was strictly regularized by capping the maximum tree depth at 5, while the total ensemble size was uniformly set to 100 estimators. The discriminative contribution of individual features was assessed using the Gini impurity reduction method, which calculates the average decrease in node impurity associated with each feature across the entire forest.

**4. Recursive Feature Elimination with Random Forest (RFE-RF)**

To further refine the predictive signature and systematically eliminate redundant variables, a hybrid Recursive Feature Elimination (RFE) strategy was coupled with the Random Forest estimator. The iterative RFE pruning process was configured with an internal Random Forest estimator (50 trees, maximum depth of 5) to continuously eliminate least important features until the top 10 most predictive variables were isolated. Subsequently, a final optimized Random Forest classifier, scaled up to 100 trees with a maximum depth of 5, was refitted exclusively on this condensed 10-feature subset to compute final classification probabilities and rank the ultimate feature importance.

**5. eXtreme Gradient Boosting (XGBoost)**

XGBoost, an advanced implementation of gradient-boosted decision trees, was applied to sequentially minimize classification errors through additive learning. To adapt to the limited cohort size and ensure stability, we applied conservative hyperparameters, including a reduced learning rate of 0.05, a maximum tree depth of 5, and an ensemble size of 100 boosting iterations. The optimization process was guided by the logarithmic loss (log-loss) evaluation metric. Feature importance was subsequently evaluated and ranked using the built-in gain metric, which represents the relative improvement in accuracy contributed by a feature across all boosting trees.

**Performance Evaluation and Feature Consistency**

To provide a standardized and comprehensive comparison, the performance of all five models—across both transcriptomic and metabolomic platforms—was assessed using pooled metrics derived from the 5-fold cross-validation. These metrics included the area under the receiver operating characteristic curve (AUC), accuracy, precision, recall, and F1-score. Furthermore, to identify the most biologically robust biomarkers, the top 10 most influential features from each algorithmic model were extracted and aggregated. This consensus approach allowed us to identify universally stable features that consistently drove the anemic phenotype across distinct mathematical paradigms.

**References**

1. Kong X, Guo Z, Yao Y, Xia L, Liu R, Song H, Zhang S: **Acetic acid alters rhizosphere microbes and metabolic composition to improve willows drought resistance**. *Sci Total Environ* 2022, **844**:157132.

2. Li C, Al-Dalali S, Zhou H, Xu B: **Influence of curing on the metabolite profile of water-boiled salted duck**. *Food Chem* 2022, **397**:133752.

3. Chen J, Li S, Zhu J, Su W, Jian C, Zhang J, Wu J, Wang T, Zhang W, Zeng F *et al*: **Multi-omics profiling reveals potential alterations in rheumatoid arthritis with different disease activity levels**. *Arthritis Res Ther* 2023, **25**(1):74.

4. Jian C, Wei L, Wu T, Li S, Wang T, Chen J, Chang S, Zhang J, He B, Wu J *et al*: **Comprehensive multi-omics analysis reveals the core role of glycerophospholipid metabolism in rheumatoid arthritis development**. *Arthritis Res Ther* 2023, **25**(1):246.

5. Zhu J, Wang T, Lin Y, Xiong M, Chen J, Jian C, Zhang J, Xie H, Zeng F, Huang Q *et al*: **The change of plasma metabolic profile and gut microbiome dysbiosis in patients with rheumatoid arthritis**. *Front Microbiol* 2022, **13**:931431.

6. Wisniewski JR, Zougman A, Nagaraj N, Mann M: **Universal sample preparation method for proteome analysis**. *Nat Methods* 2009, **6**(5):359-362.

7. Gillette MA, Satpathy S, Cao S, Dhanasekaran SM, Vasaikar SV, Krug K, Petralia F, Li Y, Liang WW, Reva B *et al*: **Proteogenomic Characterization Reveals Therapeutic Vulnerabilities in Lung Adenocarcinoma**. *Cell* 2020, **182**(1):200-225 e235.

8. Zhang H, Liu T, Zhang Z, Payne SH, Zhang B, McDermott JE, Zhou JY, Petyuk VA, Chen L, Ray D *et al*: **Integrated Proteogenomic Characterization of Human High-Grade Serous Ovarian Cancer**. *Cell* 2016, **166**(3):755-765.

9. Demichev V, Messner CB, Vernardis SI, Lilley KS, Ralser M: **DIA-NN: neural networks and interference correction enable deep proteome coverage in high throughput**. *Nat Methods* 2020, **17**(1):41-44.

10. Jones P, Binns D, Chang HY, Fraser M, Li W, McAnulla C, McWilliam H, Maslen J, Mitchell A, Nuka G *et al*: **InterProScan 5: genome-scale protein function classification**. *Bioinformatics* 2014, **30**(9):1236-1240.

11. Huang da W, Sherman BT, Lempicki RA: **Bioinformatics enrichment tools: paths toward the comprehensive functional analysis of large gene lists**. *Nucleic Acids Res* 2009, **37**(1):1-13.

12. Szklarczyk D, Gable AL, Lyon D, Junge A, Wyder S, Huerta-Cepas J, Simonovic M, Doncheva NT, Morris JH, Bork P *et al*: **STRING v11: protein-protein association networks with increased coverage, supporting functional discovery in genome-wide experimental datasets**. *Nucleic Acids Res* 2019, **47**(D1):D607-D613.

13. Gonzales PA, Pisitkun T, Hoffert JD, Tchapyjnikov D, Star RA, Kleta R, Wang NS, Knepper MA: **Large-scale proteomics and phosphoproteomics of urinary exosomes**. *J Am Soc Nephrol* 2009, **20**(2):363-379.

14. Grabherr MG, Haas BJ, Yassour M, Levin JZ, Thompson DA, Amit I, Adiconis X, Fan L, Raychowdhury R, Zeng Q *et al*: **Full-length transcriptome assembly from RNA-Seq data without a reference genome**. *Nat Biotechnol* 2011, **29**(7):644-652.

15. Wei G, Tao Y, Liu G, Chen C, Luo R, Xia H, Gan Q, Zeng H, Lu Z, Han Y *et al*: **A transcriptomic analysis of superhybrid rice LYP9 and its parents**. *Proc Natl Acad Sci U S A* 2009, **106**(19):7695-7701.
